# X-ray absorption and emission spectroscopy of N_2_S_2_ Cu(ii)/(iii) complexes†

**DOI:** 10.1039/d4dt00085d

**Published:** 2024-04-16

**Authors:** Blaise L. Geoghegan, Jessica K. Bilyj, Paul V. Bernhardt, Serena DeBeer, George E. Cutsail

**Affiliations:** a Max Planck Institute for Chemical Energy Conversion Stiftstrasse 34-36 45470 Mülheim an der Ruhr Germany george.cutsail@cec.mpg.de; b Institute of Inorganic Chemistry, University of Duisburg-Essen Universitätsstrasse 5-7 45117 Essen Germany; c Department of Chemistry, Imperial College London, Molecular Sciences Research Hub W12 0BZ London UK; d School of Chemistry and Molecular Biosciences, University of Queensland Brisbane 4072 Australia

## Abstract

This study investigates the influence of ligand charge on transition energies in a series of CuN_2_S_2_ complexes based on dithiocarbazate Schiff base ligands using Cu K-edge X-ray absorption spectroscopy (XAS) and Kβ valence-to-core (VtC) X-ray emission spectroscopy (XES). By comparing the formally Cu(ii) complexes [Cu^II^(HL1)] (HL1^2−^ = dimethyl pentane-2,4-diylidenebis[carbonodithiohydrazonate]) and [Cu^II^(HL2)] (HL2^2−^ = dibenzyl pentane-2,4-diylidenebis[carbonodithiohydrazonate]) and the formally Cu(iii) complex [Cu^III^(L2)], distinct changes in transition energies are observed, primarily attributed to the metal oxidation state. Density functional theory (DFT) calculations demonstrate how an increased negative charge on the deprotonated L2^3−^ ligand stabilizes the Cu(iii) center through enhanced charge donation, modulating the core transition energies. Overall, significant shifts to higher energies are noted upon metal oxidation, emphasizing the importance of scrutinizing ligand structure in XAS/VtC XES analysis. The data further support the redox-innocent role of the Schiff base ligands and underscore the criticality of ligand protonation levels in future spectroscopic studies, particularly for catalytic intermediates. The combined XAS-VtC XES methodology validates the Cu(iii) oxidation state assignment while offering insights into ligand protonation effects on core-level spectroscopic transitions.

## Abstract 2

The competing influences of both oxidation state and ligand protonation state in Cu(ii) and Cu(iii) Schiff base complexes are explored through copper K-edge X-ray absorption and valence-to-core emission spectroscopies along with DFT calculations.

## Graphical abstract

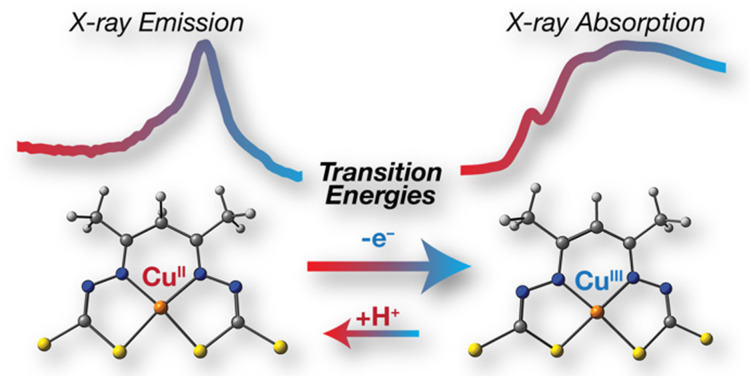

## Introduction

**Fig. 1:**
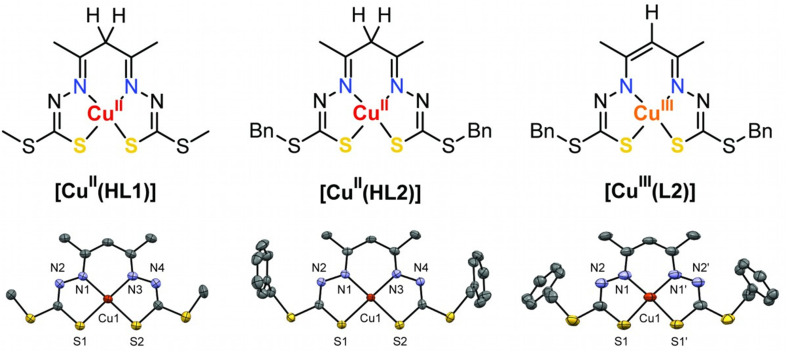
Schematic (top) and ORTEP (bottom) views of the three neutral copper complexes investigated in this work (30% probability ellipsoids shown, H-atoms omitted for clarity). Only one of the two [Cu^II^(HL1)] molecules in the asymmetric unit is shown (left, molecule A). The crystal structure of [Cu^II^(HL2)] (center) has been published previously.^9^ Bn = CH_2_C_6_H_5_.

Compared to the ubiquitous Cu(i) and Cu(ii) oxidation states, formally Cu(iii) complexes are rarely isolated and fully characterized, owing to their high reactivity and instability. Typically, stabilization of the Cu(iii) ion is achieved through the use of ligands that promote high degrees of covalency to the coordinate bonds *e.g.* N-heterocyclic carbenes (NHCs)^1,2^*and*/*or* anionic ligands such as thiolates,^3^ Schiff bases,^4^ peptides,^5^ F^−^,^6^ or CF_3_^−^_._^7,8^ Previous work^9^ demonstrated that the tetradentate (N_2_S_2_) Schiff base ligands derived from acetylacetonate and *S*-methyl or *S*-benzyl dithiocarbazate coordinate in their di- or trianionic forms to afford neutral Cu(ii) and Cu(iii) complexes, respectively (Fig. 1). This followed on from our initial discovery that the related Schiff base ligand, derived from 4,4,4-trifluoro-1-(3-thienyl)-1,3-butanedione, formed a neutral Cu(iii) complex [Cu^III^(L2)] (Fig. 1).^9^ A structurally similar bis(thiosemicarbazone) ligand was also found to be capable of forming a novel dimeric Cu(iii) complex in a comparable N_2_S_2_ coordination environment.^10^

The non-innocence of Schiff bases and the ability of these types of ligands to support both high-valent Cu(iii)^11,12^ and Fe(iv)^13,14^ oxidation states has been investigated in depth by various spectroscopic, structural and computational methods. Specifically, these dithiocarbazate Schiff bases stabilize a formally high-valent Cu(iii) oxidation state, and support for this assignment is offered in the form of electrochemical analysis and the electron paramagnetic resonance (EPR) signal observed from chemical reduction of the Cu(iii) complex to the *S* = 1/2 Cu(ii) species. As recognized previously,^11^ this does not exclude the possibility of the oxidized complex having an anti-ferromagnetically coupled Cu(ii)–L˙ structure, yielding EPR silent behavior, although, previous density functional theory (DFT) calculations of other copper Schiff base complexes have favored the *S* = 0 Cu(iii) solutions over a Cu(ii)–L˙ broken-symmetry (diradical) configuration.^11^

Although the previous studies highlighted above are consistent with an *S* = 0 Cu(iii) oxidation state description, this has yet to be spectroscopically investigated. To further characterize the electronic structure of these Schiff base-coordinated Cu(iii) systems, core-level spectroscopies such as Cu K-edge X-ray absorption (XAS) and Kβ X-ray emission (XES) offer distinct advantages to spectroscopically probe physical oxidation states. A distinction must be made between formal and physical (or spectroscopic) oxidation states. Formal oxidations of a metal ion are formulated from the removal of all ligands and counting the total integer charge left on the metal center. The physical oxidation state arises from measurables and spectroscopic observables that are interpreted in terms of a d-electron configuration and d^*n*^ count.^15^ Although the formal and physical oxidation states are often in agreement, there are numerous examples of inorganic and metalorganic complexes with radical ligand behavior, resulting in differing oxidation state interpretations. Transition metal K-edge X-ray absorption spectroscopy has long been a sensitive tool to probe the local electronic structure and valency of metal ions, coordination complexes and metalloprotein active sites.^16–18^ Historically, XAS has been used for the spectroscopic identification of oxidation state,^8,19–21^ even in high-valent Cu(iii) complexes.^2,22,23^ Pairing of XAS with Valence-to-Core (VtC) XES to probe both the unoccupied and occupied valence orbitals, respectively, is steadily becoming a more widely employed approach.^24–28^ For copper complexes and catalytic active sites, these approaches have yielded significant insight into coordination geometry,^29,30^ small molecule activation,^31^ and (debated) oxidation state assignments.^8,32,33^ Others have employed XAS to probe and distinguish between metal *vs.* ligand redox events in the presence of non-innocent ligands.^34–36^ For copper dithiocarbazate Schiff base complexes,^11^ the rare ability of these ligands to stabilize a formal Cu(iii) oxidation state is of unique interest and warrants core-level spectroscopic characterization. Recently, we demonstrated that the combination of Cu K-edge XAS and Cu Kβ valence-to-core (VtC) XES can be used to evaluate the electronic structure and physical oxidation state of Cu centers in symmetrical, highly covalent NHC and CF_3_-based, four-coordinate Cu complexes.^8^ The energies of both the XAS and VtC XES features increased incrementally with increased physical oxidation state, which was additionally supported by time-dependent density functional theory (TDDFT) calculations.

Herein, we apply this methodology to three dithiocarbazate-based complexes in either the Cu(ii) or Cu(iii) formal oxidation state (Fig. 1). The complexes studied in the present work have ligands that can be deprotonated at the carbon backbone, offering a variable degree of anionic character. Extending the combined XAS/VtC XES methodology to these dithiocarbazate complexes enables us to assess the sensitivity of core X-ray spectroscopies to changes in electronic structure when metal oxidation is accompanied by ligand deprotonation. These results are particularly pertinent to the interpretation of XAS/XES data from *in situ* experiments, where changes in metal oxidation state are often accompanied by changes in the chemical constitution of the species directly ligated to the metal center (*e.g.* in catalysis).

## Results

### Synthesis and X-ray crystallography

The complexes [Cu^II^(HL1)], [Cu^II^(HL2)] and [Cu^III^(L2)] were all prepared as described in the ESI.† Importantly, all compounds were isolated in a crystalline form. The crystal structure of [Cu^II^(HL2)] has been reported previously^9^ while the structures of [Cu^II^(HL1)] and [Cu^III^(L2)] are reported here for the first time.

The crystal structure of [Cu^II^(HL1)] (Fig. 1, left) comprises two discrete molecules, each occupying a crystallographic mirror plane in the asymmetric unit. Interestingly, they adopt different conformations based on the orientation of the *S*-methyl groups: one where the methyl groups adopt opposite orientations (molecule A) and the other where they are the same (molecule B) (Fig. S1†). It is not known whether the conformation in molecule A or molecule B, in isolation, is preferred as both are calculated to have effectively identical energies (Δ*E* = 0.14 kcal mol^−1^, Fig. S4†).

The X-ray crystal structure of [Cu^III^(L2)] is shown in Fig. 1 (right) (and Fig. S2†). The complex occupies a crystallographic two-fold axis bisecting the Cu atom and apical CH group. The *S*-benzyl groups are on opposite sides of the CuN_2_S_2_ plane. The Cu is in an approximately square planar environment with no axial contacts closer than 3.77 Å. A modest tetrahedral distortion of the CuN_2_S_2_ plane is quantified by the *τ*′_4_ parameter (*τ*′_4_ = 0.103) (which ranges from 0 for square planar complexes to 1 for tetrahedral complexes, see eqn (1) in ESI†).^37^ A more distorted geometry was found in the crystal structure of the *S*-methyl analogue [Cu^III^(L1)] (*τ*′_4_ = 0.244).^9^

The most significant structural change in [Cu^II^(HL1)] compared with [Cu^III^(L1)]^9^ and the analogous [Cu^III^(L2)] structure reported here is an elongation of the Cu–N and Cu–S coordinate bonds (Table 1), which is in-line with expectations based on the formal charge of the metal and its electronic configuration. The crystal structure of [Cu^II^(HL2)]^9^ (Fig. 1, center) exhibits close to square planar coordination geometry with no axial ligands within 3.33 Å of the metal and *τ*′_4_ = 0.065.

**Table 1:** Bond metrics as determined by X-ray crystallography and DFT optimized geometries

Bond(s)	Crystal structure			DFT		
	[Cu^II^(HL1)]^a^	[Cu^II^(HL2)]	[Cu^III^(L2)]	[Cu^II^(HL1)]	[Cu^II^(HL2)]	[Cu^III^(L2)]^b^
Lengths [Å]						
Cu–S (av)	2.25 (2.25)	2.25	2.17	2.27 (2.28)	2.27	2.21
Cu–N (av)	1.97 (1.97)	1.98	1.89	2.02 (2.02)	2.02	1.93

Angles [°]						
S1–Cu–S2	93.01 (92.15)	93.11	87.43	93.34 (93.30)	93.24	91.20
S1–Cu–N1	86.15 (86.69)	86.37	87.97	85.87 (85.89)	85.89	87.92
N1–Cu–N2	94.55 (94.73)	94.68	97.14	94.96 (95.00)	95.06	97.32
N2–Cu–S2	86.29 (86.43)	86.21	87.97	85.83 (85.81)	85.88	87.92
N1–Cu–S2	179.16 (178.84)	175.50	172.76	179.21 (179.19)	177.81	163.88
N2–Cu–S1	179.30 (178.58)	175.30	172.76	179.17 (179.11)	177.83	163.89
*τ*′_4_	0.011 (0.018)	0.065	0.103	0.011 (0.012)	0.031	0.228

a The crystal structure of [Cu^II^(HL1)] contains two formula units within the asymmetric unit, therefore the parameters for molecule A and B are given outside and inside parentheses, respectively.

b The *S* = 0 state was used for calculation of [Cu^III^(L2)] bond parameters as described in the ESI.†

Powder X-ray diffraction of all three Cu compounds matched the patterns calculated from their crystal structures derived from single crystal X-ray diffraction (Fig. S3†) thus establishing phase purity of all compounds for spectroscopic measurements.

### Theoretical structural predictions

The subtle distortion of Cu(iii) complex [Cu^III^(L2)] away from square planar is reproduced by DFT (performed in ORCA v4.2)^38–44^ only for the singlet state (*S* = 0), as well as the average Cu–S (2.17 Å) and Cu–N (1.89 Å) bond lengths, which are calculated to be only ∼0.01–0.02 Å longer than found in the crystal structure and within the expected range for deviations between theory and experiment. Attempted calculations of [Cu^III^(L2)] in the triplet (*S* = 1) state yield an effectively planar geometry (*trans* = 177.5°) and Cu–S and Cu–N bond lengths that are ∼0.1 Å longer than those observed experimentally. This disparity between the triplet state optimized structure and experiment indicates that the [Cu^III^(L2)] complex has a singlet ground state (at 190 K), and is therefore potentially either an anti-ferromagnetically coupled Cu^II^-radical system or a low-spin (LS) 3d^8^ Cu(iii) system, although the short Cu–N/S bond lengths strongly support the latter. Furthermore, the low-spin *S* = 0 d^8^ electronic configuration is typical for four-coordinate (distorted) square-planer Cu(iii) complexes with redox innocent ligands.^7,9–11,45^ An *S* = 1 observation has only yet occurred in six-coordinate Cu(iii) species, which exhibit *S* = 1 ground states due to the *O*_h_ ligand field (would also be true for *T*_d_ ligand fields).^6,46^ The singlet ground state is further supported by the single point energy of the singlet state being calculated ∼8.1 kcal mol^−1^ lower in energy than the triplet state.

### Cu K-edge X-ray absorption spectroscopy

**Fig. 2:**
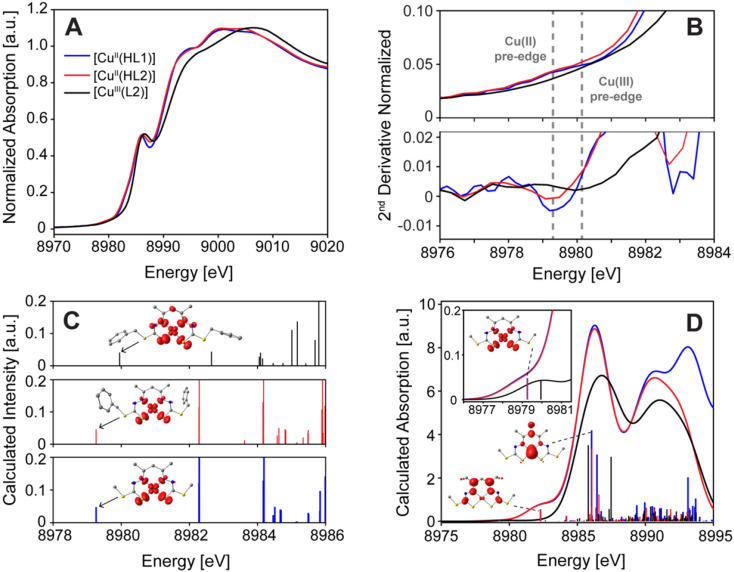
(A) Normalized Cu K-edge XAS spectra for [Cu^II^(HL1)], [Cu^II^(HL2)] and [Cu^III^(L2)]. (B) Second derivative of the pre-edge region for all three complexes and vertical grey dashed lines to guide the eye. (C) TDDFT calculated XAS transitions and difference density plots for the Cu 1s → SOMO β transition in the Cu(ii) and the Cu 1s → LUMO α + β in the Cu(iii) complexes. (D) Calculated Cu K-edge XAS spectra for [Cu^II^(HL1)], [Cu^II^(HL2)] and [Cu^III^(L2)] with one-electron transition oscillator strength plotted as sticks and transition difference density plots for selected transitions. Isosurfaces are plotted at a value of 0.005 a.u.

Cu K-edge XAS data for all three complexes were collected in transmission mode at the SAMBA beamline at the SOLEIL synchrotron (see ESI† for sample and collection details). The normalized Cu K-edge XAS spectra for [Cu^II^(HL1)], [Cu^II^(HL2)] and [Cu^III^(L2)] are shown in Fig. 2A.

Both the Cu(ii) and Cu(iii) species exhibit similar Cu K-edge XAS spectra but with some notable differences. In both Cu(ii) species the lowest energy feature is an extremely weak and broad pre-edge feature at ∼8979.3 eV corresponding to the formally electric dipole forbidden (Δ*l* = ±2) Cu 1s → 3d transition,^47^ the assignment of which is corroborated *via* TDDFT.^48^ The pre-edge features are emphasized in the second derivative spectrum shown in Fig. 2B and the TDDFT calculated transition difference densities in Fig. 2C show that these pre-edge features correspond to excitation of a Cu 1s electron (β spin) to a highly covalent Cu 3d_*x*^2^−*y*^2^_ (48.4% Löwdin population in the lowest unoccupied molecular orbital (LUMO) of the β spin manifold in the spin-unrestricted description) type molecular orbital (MO) with large amounts of metal–ligand covalency in the form of S/N p_*x*/*y*_ admixture (see below). [Cu^II^(HL1)] and [Cu^II^(HL2)] exhibit rising edge shoulders at ∼8982.8 and 8983.1 eV, respectively, which converge to well resolved and intense features at ∼8985.8 and 8986.1 eV. A second more intense rising edge feature is observed at ∼8994 eV, before maximum absorption occurs at ∼9000 eV.

In the [Cu^III^(L2)] complex, the pre-edge transition is observed at 8980.1 eV, approximately 0.8 eV higher in energy than the Cu(ii) (Fig. 2B), as expected for the higher formal oxidation state and increased *Z*_eff_,^8,22,49^ yet lower than the 8981 ± 0.5 eV range typically observed for Cu(iii) systems.^22^ The lower than expected energy of the pre-edge feature in the Cu(iii) system is related to the protonation level of the ligand, which is discussed later. The Cu(iii) pre-edge feature of ∼8980 eV stems from two excitations from the Cu 1s orbital to the LUMO in both the α and β spin manifolds, due to the closed-shell nature of the low-spin d^8^ system. From TDDFT calculations, these are also identified as transitions with Cu 3d_*x*^2^−*y*^2^_ acceptor orbital character (Fig. 2C), similar to the Cu(ii) species, albeit with significantly less d-character (28.4%). The significant decrease in the copper character of the LUMO coupled to the increased transition energy is expected for the highly charged, highly covalently bonded copper center.^8^

Unlike the Cu(ii) species however, there is no low energy shoulder at ∼8983 eV in the rising edge of the Cu(iii) species. TDDFT accurately predicts this low energy shoulder observed at ∼8983 eV in the Cu(ii) species (Fig. 2D), which arises from excitations into a ligand π* orbital with large amounts of S 3p (∼7%) and Cu (∼4%) *n*p_*z*_ orbital character. The shoulder is not observed in the Cu(iii) species due to negligible amounts of Cu *n*p admixture in the analogous acceptor MO, which is anticipated to be due to the increased *D*_2d_ distortion of the CuN_2_S_2_ first coordination sphere. It is important to note that in *D*_2d_ symmetry, the *b*_2_*x*^2^–*y*^2^ LUMO still has metal *n*p character imparted on it through admixture with the Cu 4p_*z*_ orbital (*b*_2_ symmetry),^50^ whereas in ideal *T*_d_ symmetry no such *n*p-3d mixing can occur. This transition is also calculated to be ∼0.4 eV higher in energy, a result of the increased bonding interaction energy resulting from the increase in oxidation state of the Cu center and more negative charge on the ligand.

To higher energy, the Cu(iii) species exhibits a well-resolved intense feature at ∼8986.5 eV, 0.5 eV higher in energy than the analogous feature in the Cu(ii) spectra, which is similar in magnitude to the relative energy shifts of the pre-edge features. These transitions are shown to arise from excitations into MOs with significant Cu *n*p_*z*_ character, which is significantly lower in energy than the Cu *n*p_*x*,*y*_ orbitals as there is no ligand coordination along the molecular *z*-axis (perpendicular to the CuN_2_S_2_ plane). TDDFT also accurately predicts the small energy separation of ∼0.5 eV of these features between the Cu(ii) and Cu(iii) species.

The highest energy rising edge feature in the Cu(iii) species is observed at 8996 eV, 2 eV higher than in the Cu(ii) species, while the white line is observed at ∼9006 eV, 6 eV higher in energy than the Cu(ii) species. The consistently higher energy pre-edge and edge features of the Cu(iii) species compared to the Cu(ii) species is consistent with an increased physical oxidation state^2,3,22,51,52^ on the Cu center in [Cu^III^(L2)] relative to its [Cu^II^(HL2)] analogue, demonstrating the sensitivity of Cu K-edge XAS to discriminate the Cu oxidation states even when accompanied by deprotonation of the ligand.

### Cu Kβ X-ray emission spectroscopy

Kβ mainlines (3p → 1s) typically offer spin state and/or oxidation state information for first row transition metals through the 3p–3d exchange interaction, however, metal–ligand covalency will greatly influence the 3d wavefunction, which can have pronounced effects on the final Kβ mainline spectrum.^53–56^ Splitting of the Kβ_1,3_ and Kβ′ feature is largely influenced by the 3p–3d exchange interaction in the final state, and therefore, is modulated by “dilution” of the d manifold by ligand orbital character. Increasing metal–ligand covalency acts to decrease the 3p–3d exchange integral by increasing the degree of 3d dilution, thus resulting in reduced energy separation of the Kβ_1,3_ and Kβ′ mainline features. This covalency effect on Kβ emission has been observed in first-row transitions metals such as Mn,^57^ Fe,^56,58^ Co^59^ and Ni,^53,60^ as well as Mo^61^ species. Despite the change in formal oxidation states between [Cu^II^(HL1)/(HL2)] and [Cu^III^(L2)], the Kβ (3p → 1s) mainlines are essentially superimposable, with Kβ′ and Kβ_1,3_ values of 8896.0 and 8904.7 eV, respectively (Fig. S7 and Table S1†). This is similar to what we have previously observed for other molecular copper systems.^8^

To higher energy of the Kβ mainline, VtC XES results from refilling of the copper 1s core hole by electrons from the valence orbitals.^54,62^ The transitions are significantly weaker in intensity than the mainlines and are composed of donor MOs that are a mixture of primarily ligand *n*s, *n*p and metal *n*d character. These transitions gain intensity through the relatively small percentages of metal *n*p character that mixes into the valence orbitals, which imparts greater dipole-allowed character. Due to the large amount of ligand character within the valence MO wavefunctions, VtC XES is particularly sensitive to the ligand type,^28,54,63,64^ ligand activation,^31,57,65,66^ ligand orientation relative to the metal,^27,29,67^ and can even demonstrate sensitivity to small perturbations such as protonation.^24,68,69^

**Fig. 3:**
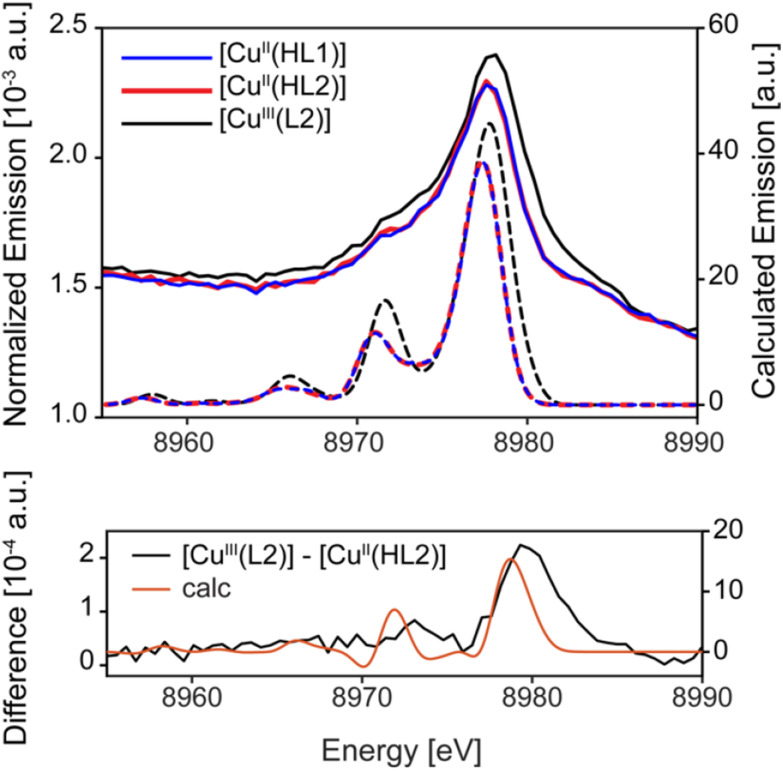
Experimental (solid) and DFT calculated (dashed) VtC spectra for [Cu^II^(HL1)], [Cu^II^(HL2)] and [Cu^III^(L2)] (top) and difference spectra for both experimental and DFT calculated VtC spectra of [Cu^III^(L2)] – [Cu^II^(HL2)]. The calculated VtC spectra for [Cu^II^(HL1)], [Cu^II^(HL2)] are effectively superimposable.

The experimental VtC XES spectra for all three complexes are shown in Fig. 3 (solid lines). The VtC XES spectra of both Cu(ii) species are effectively superimposable, with dominant Kβ_2,5_ features at ∼8977.7 eV and weaker low energy shoulders at ∼8971.3 eV. The emission intensity to higher energies of the main VtC features arise due to “double ionization” of 1s and 3d/3p electrons as the product of the incident X-ray photons having energies significantly higher than that of the Cu K-edge.^28,70^ This, in conjunction with the Cu K-edge XAS, confirms that with respect to the oxidation state and general coordination environment of the Cu center, both Cu(ii) species are indistinguishable *via* these spectroscopic methods. The Cu(iii) species also exhibits similar VtC XES spectra to the Cu(ii) species, however, the Kβ_2,5_ feature is slightly more intense and shifted by ∼0.4 eV to higher energy. The slightly higher VtC XES energy of the formally Cu(iii) complex [Cu^III^(L2)] is further supported by the large positive emission intensity at energies greater than ∼8979 eV in the difference spectra in Fig. 3 (bottom). The factors influencing these subtle differences in the VtC spectra of the Cu(ii) and Cu(iii) species are highlighted and discussed below.

**Fig. 4:**
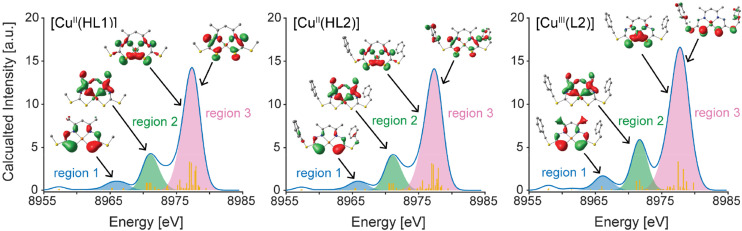
Summation of the emission intensity and representative donor MOs in three main regions of interest in the calculated VtC spectra for all complexes. MO isosurfaces are plotted at a value of 0.05 a.u.

Overall, the energies of the dominant VtC XES features across the series of complexes are determined by curve fitting the VtC XES spectra (Fig. S8†) and agree with the DFT calculated VtC XES spectra in Fig. 3. The three main regions of interest in the calculated VtC XES spectra are displayed in Fig. 4, and representative MOs are given for each. In all complexes, the VtC XES intensity arises from similar MOs for each of the three regions, respectively.

The highest intensity, dominant VtC XES feature in all three complexes (region 3) is characterized by large amounts of S 3p, N 2p and Cu *n*p orbital character as expected (Table 2). Curve fitting of the VtC XES spectra, displayed in Fig. S8,† show that this manifests experimentally as a single dominant feature at ∼8977.9 eV for the Cu(ii) species (FWHM = 2.0 eV) and 8978.0 eV for the Cu(iii) species (FWHM = 2.2 eV). The VtC emission intensity to higher energy of the VtC XES maxima is attributed to double ionization processes, which result from the use of incident X-ray photons with energies well above that of the absorption edge (Fig. S9†).^30,70,71^ While the complexes studied here are of relatively high symmetry, which will support sufficient Cu *n*p and L *n*p overlap into the in-plane copper p_*x*,*y*_ orbitals, we do note for the interest of comparison that the previously studied NHC-based complexes have approximately 25–50% greater intensity than the complexes studied here,^8^ due to their highly covalent Cu–C σ bonding interactions yielding very favorable Cu(p)–L overlap. The reduced total VtC XES intensity and changes in ligand protonation state (see below) complicate the ability of VtC XES to directly assign oxidation states of the Cu(ii) and Cu(iii) systems presented in the current study in the same way as was previously employed.^8^ However, it is apparent that although there is an overall reduction in the magnitude of the positive energy shifts in the VtC XES and Cu K-edge XAS after the oxidation of [Cu^II^(HL2)] to [Cu^III^(L2)] (simultaneous deprotonation of the ligand), the trend of increasing energy transitions in these core-level spectroscopies with increased physical oxidation state is maintained. Overall, the slight increase in VtC XES intensity for the Cu(iii) species relative to the Cu(ii) species is likely due to the subtle increase in total Cu *n*p admixture into the valence MOs, however we estimate both this admixture and the experimental intensities to be within error of one another, thus prohibiting a more quantitative assignment.

**Table 2:** Contribution of Cu d-/p-orbitals and ligand s-/p-orbitals to the calculated VtC spectra. All values are taken from the Löwdin MO population analysis using canonical orbitals

Complex	Cu d [%]	Cu p [%]	Ligand s [%]	Ligand p [%]
[Cu^II^(HL1)]^a^	9.2 (11.9)	3.4 (3.0)	13.6 (13.4)	66.6 (64.5)
[Cu^II^(HL2)]^a^	9.9 (10.8)	2.8 (2.1)	12.4 (12.0)	67.0 (66.3)
[Cu^III^(L2)]	10.2	2.8	11.8	68.6

a For Cu(ii) complexes values outside parentheses correspond to the α-spin manifold and values within parentheses correspond to the β-spin manifold.

### The influence of ligand charge

When the Cu center in [Cu^II^(HL2)] is oxidized to give the [Cu^III^(L2)] species, the ligand is also further deprotonated to its trianionic form: HL2^2−^ → L2^3−^. Here, the observed increase in energy of the XAS pre-edge feature of the Cu(iii) species relative to the Cu(ii) species is in-line with the classic analysis, which has often described this trend as an effect of increased effective nuclear charge (*Z*_eff_).^22,72,73^ This is most apparent when the formal ligand charge remains constant. Previously, we have applied this analysis to a series of organocopper complexes in formal +1, +2, +3 oxidation states where the N-heterocyclic-carbene ligand charge (and their protonation states) remains constant throughout the metal-centered oxidation steps. However, as one naturally expects, and is well demonstrated by DFT, the calculated 1s orbital energy of transition metal complexes will not only reflect the metal's *Z*_eff_, but also the ligand's charge (and protonation state). TDDFT methods are well known to exhibit large but systematic errors in calculating absolute core 1s energies and these errors affect the ability to accurately estimate *absolute* core-level spectroscopic transitions. However, the differences in the calculated *transition energies* correlate excellently to experiment. Hence, by applying a constant energy shift to TDDFT calculated metal K-edge XAS, which is extracted from a linear model of the experimental *vs.* the calculated energies, the experimentally observed transition energy trends are accurately reproduced by theory.^74^ Logically, the observed and calculated transitions are then, in part, a function of the energies of both the metal core hole and the valence orbitals, and how these respond to redox events at the metal and the ligand(s). The comparison of absolute 1s energies as a metric for assessing metal oxidation state is therefore only meaningful where the ligand remains constant in charge and character. It can be demonstrated for these CuN_2_S_2_ complexes that the inspection of the Cu 1s core energy alone is not sufficient, as naturally expected, but the experimental (and calculated) XAS and XES transitions are still an excellent reporter of oxidation state.

**Fig. 5:**
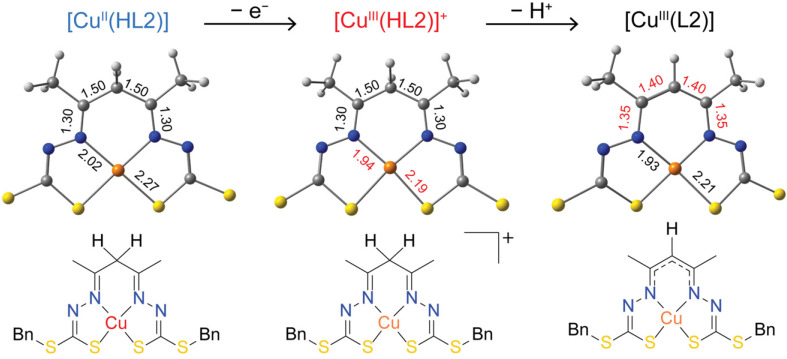
DFT optimized structures and bond lengths for [Cu^II^(HL2)], [Cu^III^(HL2)]^+^ and [Cu^III^(L2)] with benzyl groups omitted for clarity.

To further investigate the influence of the charge of the ligand (HL2^2−^*vs*. L2^3−^), DFT calculations were firstly performed on the computationally-generated one-electron oxidation product [Cu^III^(HL2)]^+^ from [Cu^II^(HL2)], which retains the protonated form of the ligand and is formally metal oxidized (d^8^, *S* = 0), and secondly on the one-electron reduction product [Cu^II^(L2)]^−^ from [Cu^III^(L2)], which retains the deprotonated form of the ligand and is formally metal reduced (d^9^, *S* = 1/2). The latter models the electronic situation encountered experimentally for the electrochemical reduction of [Cu^III^(L2)] in aprotic solvent which generates anionic Cu(ii) species including the analogous compounds [Cu^II^(L1)]^−^ and [Cu^II^(ttfasbz)]^−^.^9,11^ The DFT optimized structure of [Cu^III^(HL2)]^+^ exhibits bond metrics that resemble the formally Cu(iii) complex [Cu^III^(L2)], including shortened Cu–N_av_ (1.94 Å) and Cu–S_av_ (2.19 Å) bond lengths and a larger *D*_2*d*_ distortion of the CuN_2_S_2_ first coordination sphere (*τ*′_4_ = 0.122) compared to [Cu^II^(HL2)] (*τ*′_4DFT_ = 0.031), as expected for a more highly oxidized Cu(iii) complex (Table S2†). However, unlike the deprotonated [Cu^III^(L2)] complex, whose ligand exhibits a greater negative charge delocalized across the backbone, [Cu^III^(HL2)]^+^ appears to exhibit ligand characteristics that are nearly identical to [Cu^II^(HL2)] (Fig. 5). The shorter Cu–L bonds for [Cu^III^(HL2)]^+^ compared to [Cu^II^(HL2)], and a greater distortion of the CuN_2_S_2_ first coordination sphere leads to three possible interpretations: (a) the ligand donor is more negatively charged and the Cu center remains unchanged, (b) the Cu center is more positively charged and the ligand remains unchanged, or (c) the ligand donor is more negatively charged and the Cu center is more positively charged. All taken together, both the ligand structure and the Cu–L structural parameters alongside the calculated pre-edge energies, interpretation (b) provides the closest agreement, suggesting the oxidation event is mostly restricted to the Cu center (Fig. 5). Similarly, the DFT optimized structure of [Cu^II^(L2)]^−^ exhibits Cu–N_av_ (1.98 Å) and Cu–S_av_ (2.29 Å) bond lengths and a planar CuN_2_S_2_ first coordination sphere (*τ*′_4_ = 0.022) that resembles the Cu(ii) species [Cu^II^(HL2)], but with a deprotonated ligand backbone and ligand bond lengths that resemble [Cu^III^(L2)] (Table S2†).

**Fig. 6:**
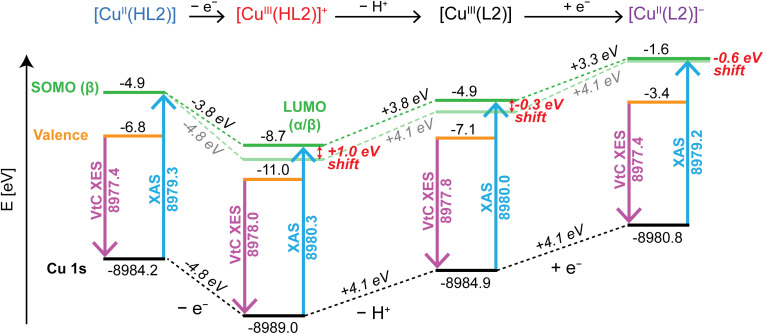
Comparison of the calculated Cu Kβ VtC XES (purple arrow) and Cu K-edge XAS (blue arrow) for [Cu^II^(HL2)], [Cu^III^(HL2)]^+^, [Cu^III^(L2)] and [Cu^II^(L2)]^−^, and their calculated energy level diagrams. The as-calculated SOMO/LUMO energies are displayed in the dark green color, whereas the light green line displays the constant SOMO/LUMO energy trend relative to [Cu^II^(HL2)] using the same energy change of the 1s. The effective energy shift of the pre-edge transition energy for each step is highlighted in red.

In the calculated Cu K-edge XAS, the pre-edge feature of [Cu^III^(HL2)]^+^ (8980.3 eV) is 0.3 eV higher in energy than [Cu^III^(L2)] (8980.0 eV) and 1 eV higher than [Cu^II^(HL2)] (8979.3 eV) (Fig. 6, and S10B†). This ∼1 eV shift in the pre-edge feature between [Cu^II^(HL2)] and [Cu^III^(HL2)]^+^ is in-line with other examples of Cu(ii)/Cu(iii) redox isomers^8,22^ and suggests that deprotonation of [Cu^III^(HL2)]^+^ to form [Cu^III^(L2)] modulates the energy of the XAS pre-edge transition (Fig. S10B†). The calculated VtC XES emission energy of the main Kβ_2,5_ feature for [Cu^III^(HL2)]^+^ (8978.0 eV) (Fig. S10A†) is 0.6 eV higher than [Cu^II^(HL2)] (8977.4 eV) and 0.4 eV higher than [Cu^III^(L2)] (8977.8 eV), following the same trend as the calculated pre-edge XAS transitions, albeit with smaller magnitude. This demonstrates that when metal oxidation is accompanied by ligand deprotonation, such as in the [Cu^II^(HL2)] → [Cu^III^(L2)] transformation, the increase in energy of both the pre-edge absorption and Kβ_2,5_ XES features are diminished relative to a pure one-electron oxidation event. This may suggest that the fully deprotonated (L2)^3−^ ligand is capable of donating some electron density back to the Cu center *via* an increase in metal–ligand d–π orbital overlap, once again highlighting the intrinsic link between the XAS/XES transition energies and the metal *Z*_eff_. Overall, the XAS pre-edge, edge and Kβ_2,5_ XES energies of [Cu^III^(L2)] still shift to higher energy compared to [Cu^II^(HL2)], which is consistent with an increased physical oxidation state. Therefore, the formal Cu(iii) oxidation state assignment of the [Cu^III^(L2)] complex is upheld. Conversely, the theoretical [Cu^II^(L2)]^−^ complex exhibits a nearly identical VtC XES emission spectrum and pre-edge energy to [Cu^II^(HL2)], but the characteristic ∼8982.8/8983.1 eV shoulder feature seen in the rising edge of the [Cu^II^(HL1)]/[Cu^II^(HL2)] complexes is nonexistent (Fig. S10B†). This shows that in the Cu(ii) oxidation state, only the rising edge features in the Cu K-edge XAS appear sensitive to the changes in ligand protonation level, and that this change between (HL2)^2−^ and (L2)^3−^ influences the energy of the VtC XES features more dramatically in highly oxidized Cu(iii) systems. This is in agreement with our previous studies on Cu-NHCs,^2,8^ and emphasizes the importance of considering changes in the ligand's chemical/electronic composition when interpreting VtC XES data.

The increased donation of electron density to the Cu(iii) center in the fully deprotonated form [Cu^III^(L2)] is best demonstrated by the calculated energy level diagram (Fig. 6). Simultaneous ligand deprotonation and metal oxidation ([Cu^II^(HL2)] → [Cu^III^(L2)]) significantly raises the Cu 1s orbital energy relative to the metal-only oxidized Cu(iii) species ([Cu^II^(HL2)] → [Cu^III^(HL2)]^+^), showing how the increased negative charge of the (L2)^3−^ ligand counteracts the expected stabilization of the Cu 1s orbital upon metal-centered oxidation compared to (HL2)^2−^. This modulation of the Cu 1s orbital energy emphasizes the inextricable link between the Cu *Z*_eff_ and the XAS/XES transition energies, which are typically incremental and predictable when the ligand charge (and geometry) remains constant^8^ but can be significantly impacted when it does not. The −0.3 eV shift of the pre-edge transition when going from [Cu^III^(HL2)]^+^ to [Cu^III^(L2)] likely occurs due to subtle changes in electron donating effects concomitant with increasing total negative charge from deprotonation. This, in turn, reduces the energy separation between LUMO/valence MOs and the Cu 1s orbital when the Cu *Z*_eff_ is at a more positive energy, manifesting spectroscopically as decreased energies of the XAS and VtC XES features. Nonetheless, both formally Cu(iii) species in Fig. 6 exhibit large and clear shifts of their pre-edge energies relative the Cu(ii) species.

The ideas of ligand influences on the core transition energies were previous investigated by Kubin *et al.* by calculating the *in silico* reduction of Mn^III^(acac)_3_ at a fixed geometry and the relaxed geometry.^75^ The reduction overall led to a −1.4 eV shift in the calculated L-edge transition energy, reflecting the valency change, where the difference in the fixed and relaxed geometries only counteracted this calculated shift by +0.2 eV. Therefore, the observed transition energy change was most dependent on the metal center's oxidation state. We can apply a similar observation to our *in silico* experiments (Fig. 5 and 6), where we do see a large positive shift in the copper K-edge pre-edge energy with the *in silico* oxidation from [Cu^II^(HL2)] to [Cu^III^(HL2)]^+^. However, this shift is slightly counteracted, by 0.3 eV with the ligand deprotonated. We infer the slight changes in the Cu–L distances with the more negatively charged ligand counteracts the initial positive *in silico* calculated metal oxidation transition energy. This is well in-line with the 0.2 eV influence Kubin *et al.* observed for the minor geometry differences.

## Summary

The Cu K-edge XAS and Kβ VtC XES spectra for the Cu(ii) complexes [Cu^II^(HL1)] and [Cu^II^(HL2)] and Cu(iii) complex [Cu^III^(L2)] have been studied in order to probe the role of ligand charge on the transition energies using these core X-ray spectroscopies. Typically, metal K-edge XAS has been used as a key indicator of the metal ion oxidation state, and recently, we showed that this is also true for the Kβ VtC XES transitions when the ligand environment is preserved between metal oxidation states.^8^ In the current study, the [Cu^III^(L2)] complex exhibits only subtle variations in its Kβ VtC XES and more pronounced differences in the Cu K-edge XAS when compared to the Cu(ii) analogs. The changes in transition energies are shown *via* DFT calculations to be due to the competing effects of simultaneous ligand deprotonation and metal oxidation, where the protonation state of the ligand has the ability to slightly modulate the observed XAS transition energy. *In silico* oxidation of the [Cu^II^(HL2)] system to give [Cu^III^(HL2)]^+^ (Fig. 6) demonstrates clearly how the Cu(iii) center in [Cu^III^(L2)] is stabilized through increased charge donation from a more negatively charged ligand, which works to provide negative electron density that counteracts the effects of an increased effective nuclear charge on the Cu center. This effect is particularly noticeable in the energies of the Cu 1s orbitals, which are heavily modulated by the total negative charge on the ligand. These results show that increasing the negative charge of the ligand from 2^−^ → 3^−^ simultaneous to the Cu(ii) → Cu(iii) oxidation leads to a reduced *net decrease* in energy of the Cu 1s orbital relative to the *valence MOs* compared to when the ligand remains as a dianion, meaning the expected +1.0 eV increase in energy of the Cu K-edge XAS pre-edge/edge and Kβ VtC XES transitions are actually observed to be *ca.* +0.7 eV for Cu(iii) *vs.* Cu(ii) centers. Despite this, there is a clear and significant shift to higher energies in both the XAS and VtC XES experiments upon metal oxidation, even with the increased negative charge of the ligand in the [Cu^II^(HL2)] → [Cu^III^(L2)] transformation. It is important to note however, that these core X-ray spectroscopies do not directly probe the energy of the Cu 1s orbital, but rather it's influence on the relative energy separation of the Cu 1s from the valence band and unbound states. Hence, the work presented in the current study highlights two key points: (1) the chemical and electronic structure of the ligand should be diligently scrutinized when employing XAS/VtC XES to assign metal oxidation states and (2) despite electron shielding effects playing a notable role in modulation of the XAS/VtC XES transition energies, the combination of these spectroscopies remains a reliable method for determining the oxidation state of metal centers even in dynamic/flexible ligand environments.

The XAS data herein supports a clear redox innocent role of the Schiff base ligand for these copper complexes. The observed spectroscopic transitions and their shifts in energy are consistent with metal-centered redox and correlate to the formal oxidation state. The influence of the protonation level of the ligand, albeit small, is critical for the future employment of XAS and VtC XES spectroscopy to catalytic intermediates. For instance, numerous copper water oxidation catalysts (WOC),^76–79^ with and without non-innocent ligands, and copper monooxygenase mechanisms^80–82^ invoke potential ‘high-valent’ intermediates. Many of these ligands utilize various carboxylates or amino groups as potential hydrogen-atom donors in the case of WOC and copper proteins, respectively. Therefore, understanding the sensitivity of these core-level spectroscopic techniques to both oxidation and protonation state of the coordinating ligand will be necessary for accurate analysis in such future spectroscopic studies.

In conclusion, we have shown that the analysis of a combined metal K-edge XAS–Kβ VtC XES methodology supports the previously assigned Cu(iii) oxidation state of [Cu^III^(L2)], while simultaneously providing deep insight into the influences of ligand protonation level on the energies of core X-ray spectroscopic transitions.

## Author contributions

GEC, SDB and PVB conceived and supervised the study. JKB synthesized all compounds. PVB determined the XRD structures of the complexes in this work. BLG and GEC collected experimental X-ray spectroscopic data, carried out data processing and performed quantum chemical calculations. BLG and GEC wrote the manuscript. All authors discussed and commented on the manuscript.

## Data availability

• Crystallographic data for [Cu^III^(L2)] and [Cu^II^(HL1)] has been deposited in the CCDC under 2204793 and 2204794† and can be obtained from https://www.ccdc.cam.ac.uk.

• The *xyz* coordinates used for quantum chemical calculations within this article have been uploaded as part of the ESI.†

• Cu Kβ XES and K-edge XAS data are available in ASCII format *via* the Edmond Open Research Data Repository of the Max Planck Society at https://doi.org/10.17617/3.UXUIMI.

## Conflicts of interest

There are no conflicts of interest to declare.

## Supplementary Material

DT-053-D4DT00085D-s001

DT-053-D4DT00085D-s002
