# Supplementary material for: X-ray absorption and emission spectroscopy of N2S2 Cu(ii)/(iii) complexes
Source: Dalton Trans. 2024 Apr 16;53(18):7828–38. doi: 10.1039/d4dt00085d (PMC11075465; doi:10.1039/d4dt00085d)
Supplement: DT-053-D4DT00085D-s001 [file DT-053-D4DT00085D-s001.pdf]

Electronic Supplementary Material (ESI) for Dalton Transactions.
This journal is © The Royal Society of Chemistry 2024

X-ray Absorption and Emission Spectroscopy of N_2S_2 Cu(II)/(III) Complexes

Blaise L. Geoghegan,[a,b,c] Jessica K. Bilyj,[d] Paul V. Bernhardt,[d] Serena DeBeer,[a] George E. Cutsail III[a,b]*

[a] Max Planck Institute for Chemical Energy Conversion, Stiftstrasse 34–36, 45470 Mülheim an der Ruhr, Germany

[b] Institute of Inorganic Chemistry, University of Duisburg-Essen, Universitätsstrasse 5–7, 45117 Essen, Germany

[c] Department of Chemistry, Imperial College London, Molecular Sciences Research Hub, W12 0BZ, London, UK

[d] School of Chemistry and Molecular Biosciences, University of Queensland, Brisbane 4072, Australia

email: george.cutsail@cec.mpg.de

Supporting Information

Experimental

Synthesis

The free ligands H_3L1 and H_3L2 were prepared as previously described.[1] Both ligands are present in their cyclic pyrazoline form, which ring-open upon metal coordination (Scheme S1). The solution chemistry of [Cu^{II}(HL1)], [Cu^{II}(HL2)] and [Cu^{III}(L2)] has been reported and the crystal structure of [Cu^{II}(HL2)] has been published.[1] Synthetic routes to these complexes on a preparative scale are described for the first time below.

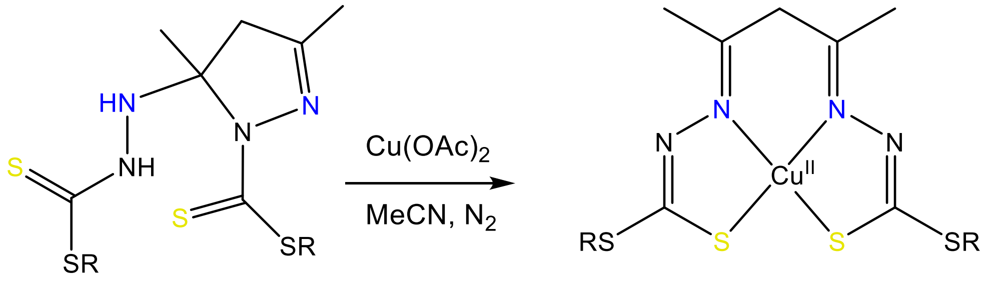

H_3L1 R = Me
H_3L2 R = Bn

[Cu(HL1)] R = Me
[Cu(HL2)] R = Bn

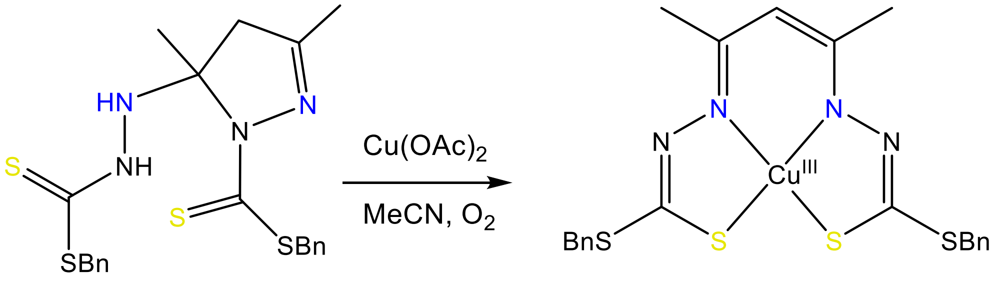

H_3L2

[Cu(L2)]

Scheme S1. Synthetic approach leading to either Cu(II) or Cu(III) complexes.

[Cu^{II}(HL1)] and [Cu^{II}(HL2)]

Separate solutions of H_3L1 (176 mg, 570 μmol) (or an equivalent amount of H_3L2) and copper acetate monohydrate (114 mg, 570 μmol) were prepared in 15 mL of deoxygenated MeCN within a Belle Technology glove box (O_2 < 20 ppm) with gentle warming. Upon combining the two solutions, an immediate colour change to brown was observed. Within 10 minutes, light brown needles began to crystallise and upon cooling (in the glove box) precipitation was complete. The crystalline products were collected by filtration (under aerobic conditions) and washed with a minimum amount of cold EtOH. The yield of [Cu^{II}(HL1)] was 48% while [Cu^{II}(HL2)] was 49%.

An identical crystalline product could also be obtained using the same procedure above except with distilled, oxygen free DMF and 3 equivalents of triethylamine added upon immediate complexation. However, crystallisation took several days rather than minutes in MeCN.

[Cu^{III}(L2)]

In individual beakers, H_3L2 (100 mg, 217 μmol) and copper acetate monohydrate (43 mg, 217 μmol) were each dissolved in 15 mL of MeCN (or DMF) with gentle heating and stirring. The solutions were combined and stirred for 10 min with gentle warming. Stirring and heating was discontinued, and the solution was allowed to slowly evaporate on the bench over one week. Brown crystals that formed were filtered off from the surrounding solvent and washed with cold ethanol and dried under vacuum. The yield was approximately 50%.

Phase purity of these crystalline compounds was established by a combination of single crystal and powder X-ray diffraction (see below).

Structure

Single Crystal X-ray Diffraction

Single crystal X-ray diffraction (XRD) data for [Cu^{III}(L2)] were collected on an Oxford Diffraction Gemini Ultra S diffractometer using Cu-Kα radiation (1.54184) at 190 K. Single crystal XRD data for [Cu^{II}(HL1)] were collected at the Australian Synchrotron at a temperature of 100 K. Both structures were solved with SHELXT and refined with SHELXL.[2] All calculations were carried out within the WinGX interface. Data in CIF format have been deposited with the CCDC (numbers 2204793 and 2204794).

Molecular structures of [Cu^{II}(HL1)] and [Cu^{III}(L2)] are shown in Figures S1 and S2 created with Mercury.[3] There are two molecules of [Cu^{II}(HL1)], each occupying a crystallographic mirror plane, that comprise the asymmetric unit. They differ in the dispositions of their S-methyl groups (Figure S1). In the structure of [Cu^{III}(L2)] (Figure S2) the complex is situated on a two-fold axis.

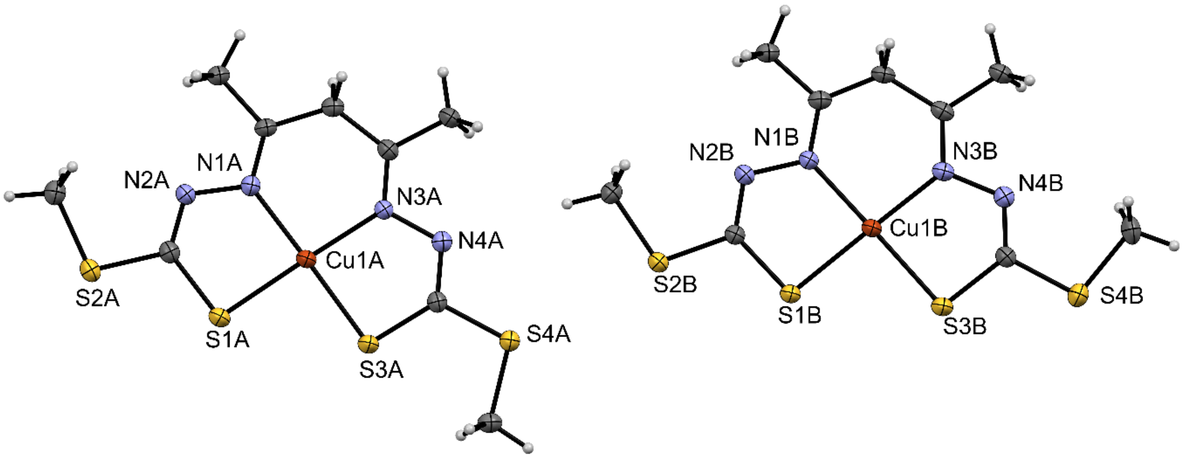

Figure S1 ORTEP view of the two molecules of [Cu^{II}(HL1)] in the asymmetric unit with 30% probability ellipsoids shown (CCDC 2204794).

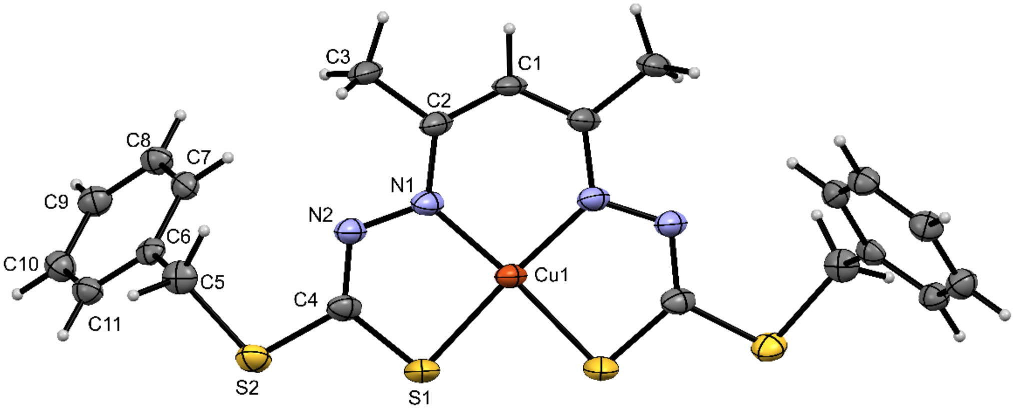

Figure S2 ORTEP view of [Cu^{III}(L2)] with 30% probability ellipsoids shown (CCDC 2204793).

Powder X-ray Diffraction

Powder XRD data were collected on crystalline samples ground to a fine powder and mounted in quartz capillaries. The data were collected an Oxford Diffraction Gemini Ultra S diffractometer using Cu-Kα

radiation (1.54184 Å) by a ϕ-scan within the range 2θ = ±40° at 190 K. The theoretical PXRD patterns were calculated with Mercury.[3] These are shown in Figure S3.

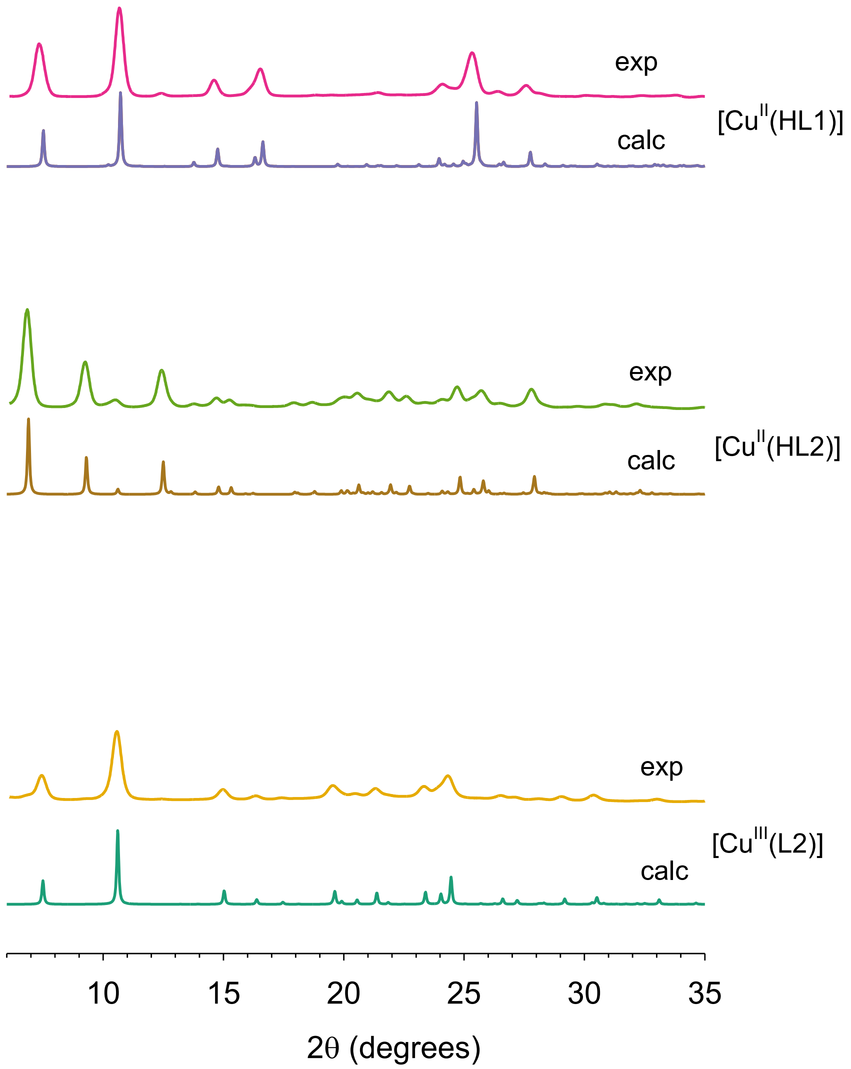

Figure S3 Experimental and calculated powder XRD patterns for [Cu^{II}(HL1)], [Cu^{II}(HL2)] and [Cu^{III}(L2)] (λ = 1.54184 Å, T = 190 K).

X-ray Absorption Data Collection

Samples for X-ray absorption spectroscopy were prepared as solid mixtures in boron nitride containing 2% Cu (w/w). The mixtures were finely ground and tightly packed into 1 mm aluminum sample cells sealed with 38 μm Kapton tape.

All three samples were measured in transmission mode at room temperature and ambient conditions at the SAMBA beamline of the Soleil synchrotron under ring conditions of 3 GeV and 500 mA. Incident X-ray radiation was monochromatized by using a Si(220) sagittal focusing monochromator between

two bendable cylindrical mirrors. The incident energy was calibrated by using a Cu foil calibration standard and assigning the energy of the first inflection point to 8980.3 eV. Twenty scans were measured and averaged using in-house developed MATLAB R2019b scripts and all further data processing was conducted using the Athena program for X-ray spectroscopic data processing.[4] The background was accounted for by fitting a polynomial to the pre-edge region and subtracting this polynomial from the entire spectrum. All spectra were normalized by fitting a flattened polynomial to the post-edge region of the spectra and normalizing the edge jump at 9000 eV to 1.0. No spectral changes due to photodamage were observed after up to 360 10 s scans on a single sample spot (beam size = 2000 x 300 μm {width x height}).

X-ray Emission Data Collection

Samples for XES experiments, all samples were measured in the solid state at room temperature. The pure solids were ground to a fine powder and packed into 0.5 mm thick aluminum sample holders and sealed with 38 μm Kapton tape.

XES data were measured at the PINK beamline (during beamline commissioning) at BESSY II.[5,6] A considerable gain in fluorescence signal could be obtained by using a multilayer monochromator (~100 eV band pass), and the energy of the incident beam was 9.5 keV. The beam size on the sample was 30 μm × 500 μm (vertical × horizontal. All spectra were collected using an in-house-designed energy-dispersive von Hámos spectrometer with a vacuum sample chamber environment (5 mbar working pressure). The analyzer was set up in a vertical dispersion direction, taking advantage of the small vertical beam size to improve the energy resolution. A Si(444) crystal with a bending radius of 250 mm dispersed incoming fluorescence radiation onto an Eiger detector with a 75 μm × 75 μm pixel size. The 80 mm x 40 mm detector chip accepted fluorescent radiation reflected from the crystal analyzer under 68.0º-60.7º Bragg angles that corresponded to an energy window of 8530-9065 eV. Calibration of the energy scale was achieved by measuring reference X-ray emission lines of anhydrous CuCl in the same instrumental configuration and setting the energy of the $K\beta_{1,3}$ mainline feature to 8904.7 eV and VtC feature to 8977.4 eV.[6]

Cu Kβ XES damage scans and assessments were performed for all samples. In all cases, the data were collected in continuous sample motion. Samples were scanned at a rate of 100 μm/s, resulting in a total exposure time of 0.50 s/pass and the total exposure was between 6 and 10 s per scan of the sample. None of the samples exhibited damage or changes in the Kβ emission spectra within the measurement scan periods. XES data were processed and fit using in-house developed MATLAB scripts and the integrated intensity of the Kβ main line was set to 1.0.

Density Functional Theory

Quantum chemical calculation were performed in the ORCA electronic structure package v4.2.[7,8] Starting coordinates for quantum chemical calculations were taken directly from the crystal structures for all three complexes and their geometries were optimized using BP86/def2-TZVP.[1,9,10] The structures of $[Cu^{III}(HL2)]^{+}$ and $[Cu^{II}(L2)]^{-}$ were obtained through the *in silico* one-electron oxidation or reduction of $[Cu^{II}(HL2)]$ or $[Cu^{III}(L2)]$, respectively, and allowing the geometry to relax. Both TDDFT and DFT calculations were carried out at the TPSSh[11,12] level of theory, using def2- variants of Alrich's all-electron Gaussian triple-ζ valence polarized recontracted scalar relativistic basis set (ZORA-def2-TZVP)[10] on all atoms and the AutoAux basis option for ORCA.[13] The calculations employed the resolution of identity (RI-J) algorithm for the computation of the Coulomb terms and the 'chain of spheres exchange' (COSX) algorithm for the calculation of the exchange terms.[14] In all calculations a tight self-consistent field (SCF) convergence threshold was chosen. An increased grid was used during the SCF iterations (Grid4), and for the final energy evaluation after SCF convergence Grid7 was used. The zeroth-order regular approximation (ZORA) was employed for all XAS and XES calculations to account for scalar relativistic effects.[15,16] A 2.5 eV broadening was applied to calculated XAS spectra and a 2.0 eV broadening was applied to calculated XES spectra. A corrective +0.5 eV energy shift was applied to the calculated XAS spectra, whereas the XES spectra were shifted +0.2 eV. Vibrational frequencies were calculated for all optimized structures and the absence of imaginary modes alongside six 0 cm^{-1} modes confirmed that the true minima were obtained in all cases. Exchange coupling constants J for the 1 electron oxidized metal-radical species Cu^{II}-$HL2^{\bullet}$ were estimated using the broken-symmetry DFT methodology (BS-DFT) at the TPSSh/def2-TZVP level of theory. Initial guesses for the broken-symmetry calculations were created using the 'BrokenSym' feature of ORCA, which exchanges the α and β spin blocks of the density on the specified centers after converging the high-spin wave function.

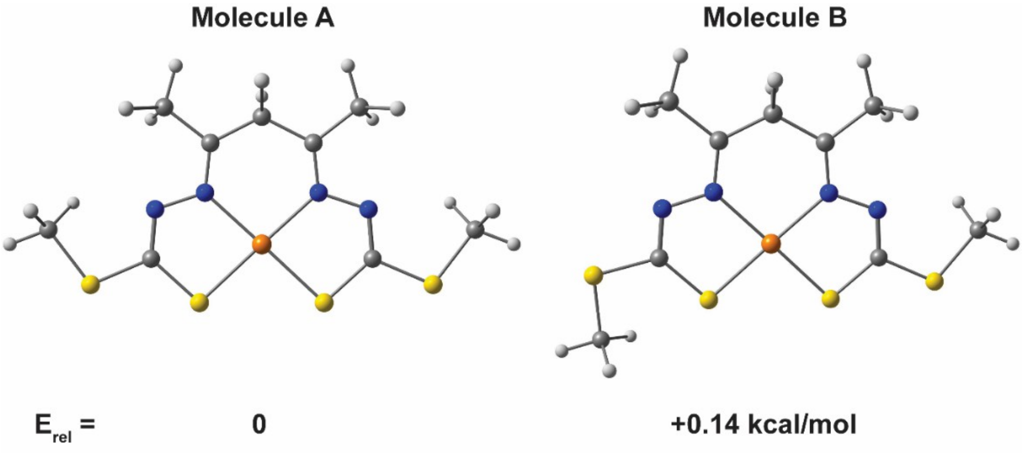

Figure S4 DFT optimized structures for $[Cu^{II}(HL1)]$ molecule A and B and their associated relative single point energies.

Calculation of τ_4' parameters

$$\tau_4' = \frac{\beta-\alpha}{360-\theta} + \frac{180-\beta}{180-\theta} \quad (1)$$

Where β is the largest L-M-L angle in the first coordination sphere, α is the second largest L-M-L angle in the first coordination sphere and θ is the ideal tetrahedral L-M-L angle 109.5°. For ideal tetrahedral geometry $\beta = \alpha = 109.5°$ and $\tau_4' = 1$. For ideal square-planar geometry $\beta = \alpha = 180°$ and $\tau_4' = 0$.[17,18]

X-ray Absorption Spectroscopy

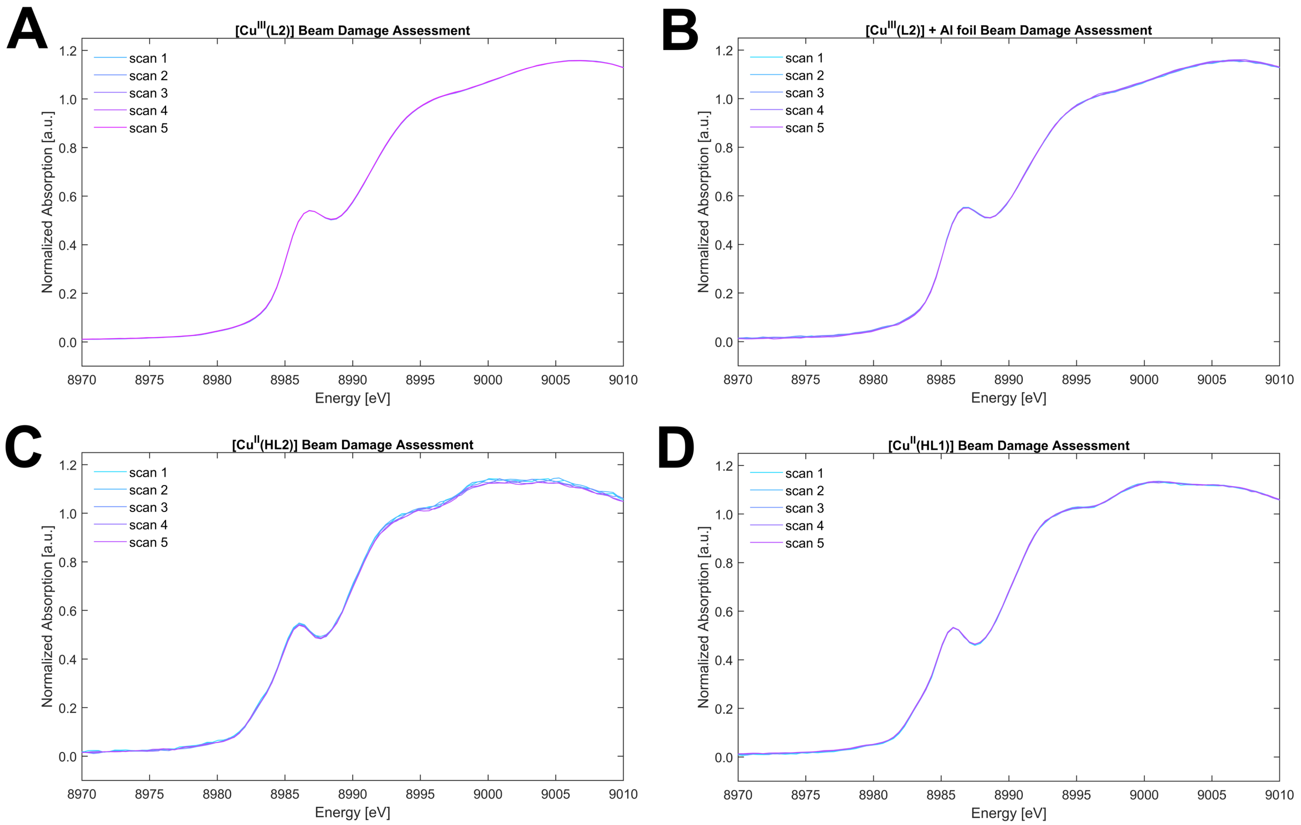

Figure S5 Beam damage assessment Cu K-edge XAS spectra obtained on single sample spots of [Cu^{III}(L2)] in unattenuated beam (**A**), [Cu^{III}(L2)] with 250 μm Al foil filter (**B**), [Cu^{II}(HL2)] in unattenuated beam (**C**) and [Cu^{II}(HL1)] in unattenuated beam (**D**).

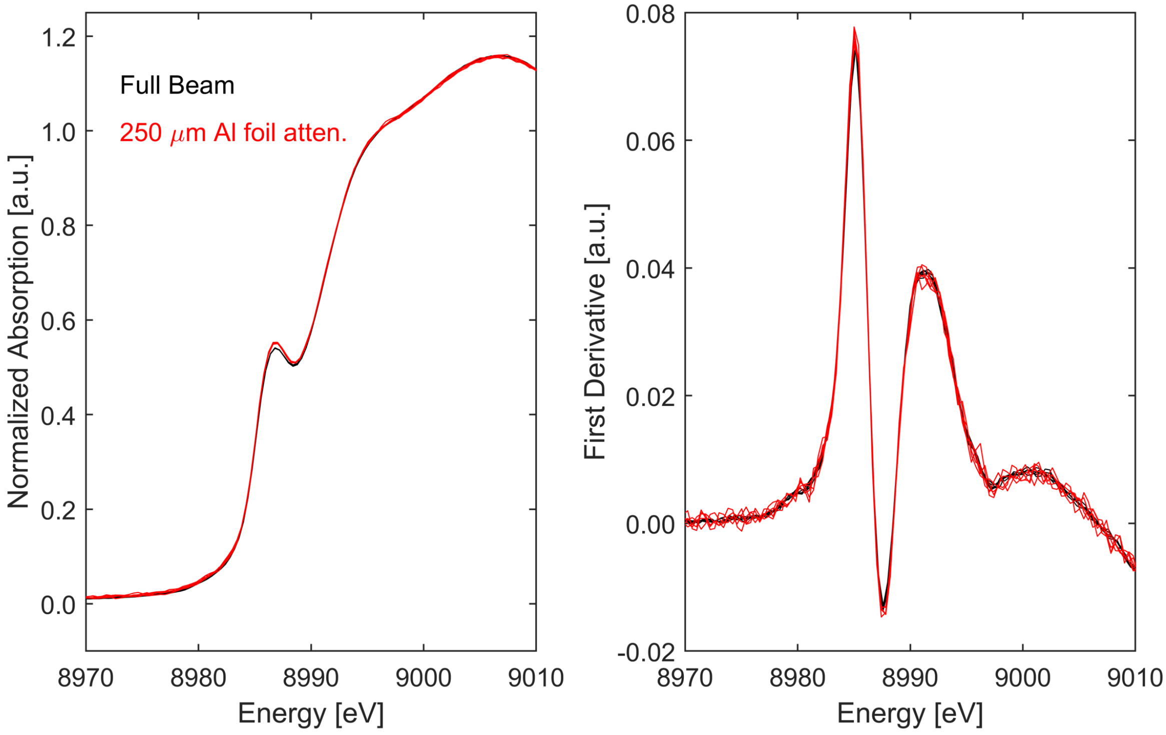

Figure S6 Comparison of Cu K-edge XAS spectra for [Cu^{III}(L2)] collected with unattenuated beam (black) and with the beam attenuated by a 250 μm Al foil filter (red).

X-ray Emission Spectroscopy

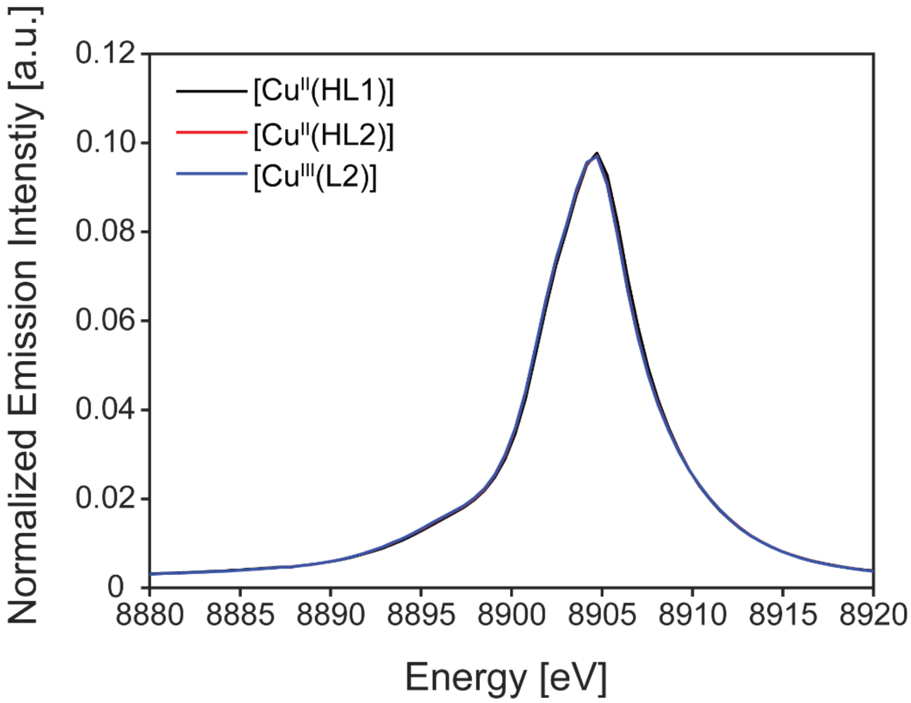

Figure S7 Cu Kβ mainlines for [Cu^{II}(HL1)], [Cu^{II}(HL2)] and [Cu^{III}(L2)].

Table S1 Energies of the Kβ' and $K\beta_{1,3}$ features, and their separation, for all complexes considered in the present study.

	**Kβ' (eV)**	**$K\beta_{1,3}$ (eV)**	**Δ*E* (eV)**
$[Cu^{II}(HL1)]$	8896.0	8904.7	8.7
$[Cu^{II}(HL2)]$	8896.0	8904.7	8.7
$[Cu^{III}(L2)]$	8896.0	8904.7	8.7

The first moment of the VtC spectrum can be used to analyze subtle shifts in the average energy of the whole spectrum and is calculated *via*

$$M_j = \frac{\sum_j (E_j I_j)}{\sum_j I_j} \qquad (2)$$

where E_j is the energy of a spectral data point and I_j is its intensity.

Fitting the experimental VtC spectra show that all three complexes have a single dominant $K\beta_{2,5}$ feature centered at ~8979.9 eV for Cu(II) and 8980.0 eV for Cu(III) (Figure S7). This dominant feature has a FWHM of 2.0 eV for the Cu(II) species but increases to 2.2 eV in the Cu(III) species, concomitant with the subtle increase in intensity (5.2 a.u. for Cu(II) and 6.0 a.u. for Cu(III). A shoulder is present to the lower energy side of the main feature in all three complexes, brought about by two broader emission bands of lower intensity.

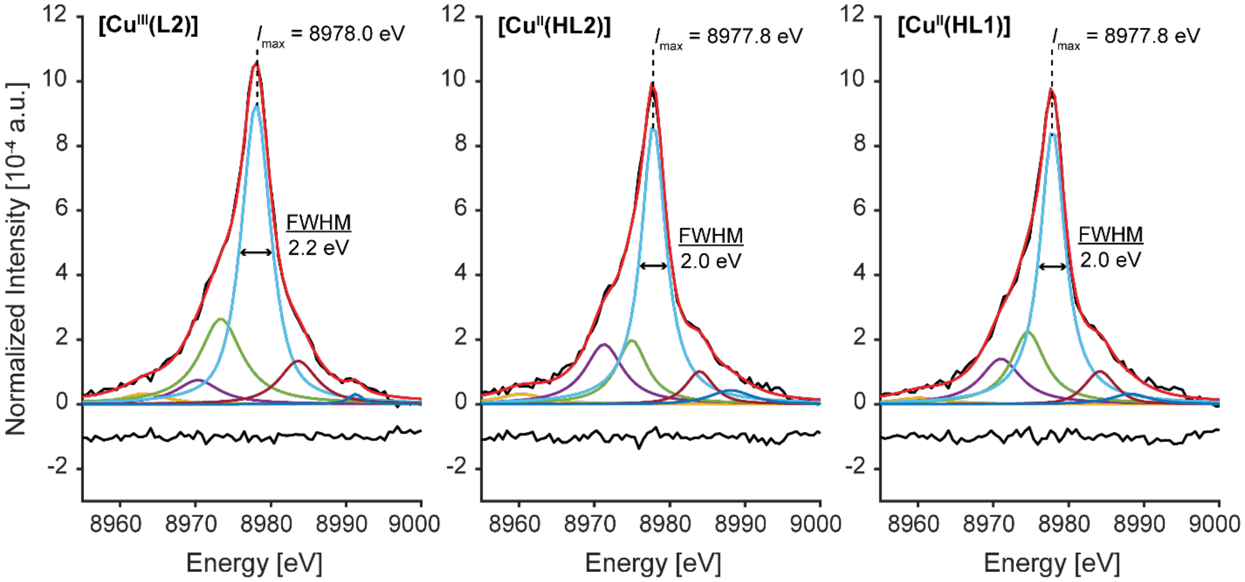

Figure S8 Background subtracted VtC spectra (black) with individual fitted Gaussian bands (colored) and their sum (red). Residual intensity (experiment – fit) are shown in black below the individual VtC spectra.

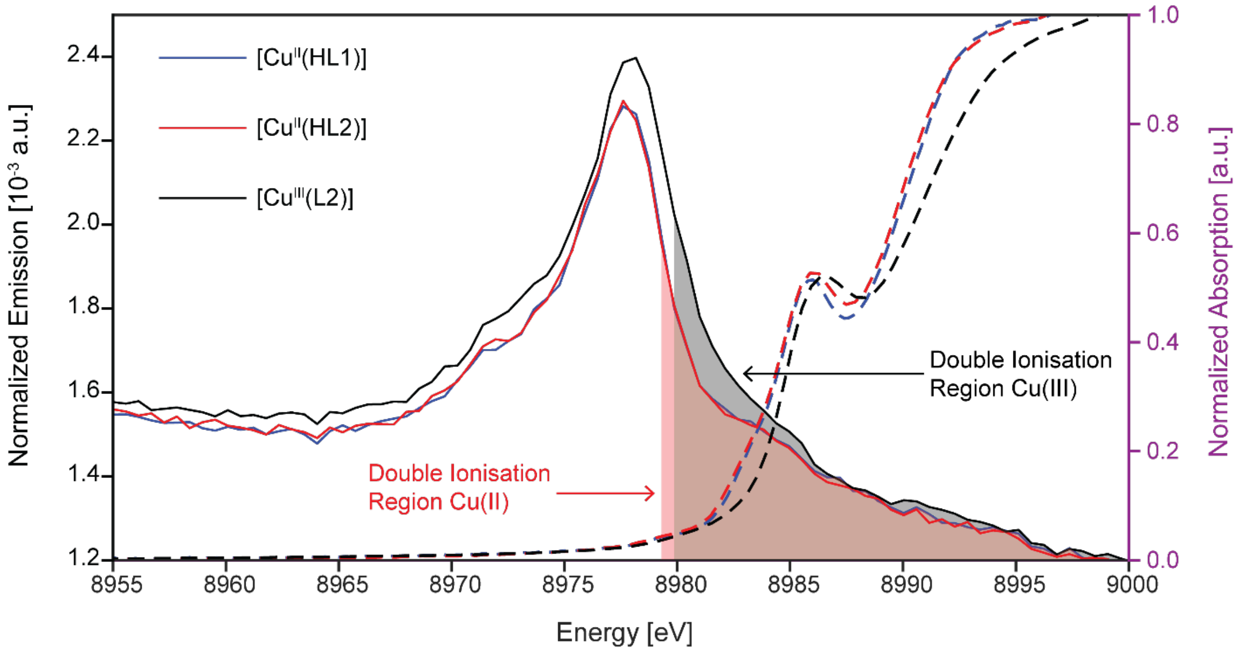

Figure S9 VtC XES spectra (solid lines) and Cu K-edge XAS spectra (dashed lines) for [Cu^{II}(HL1)], [Cu^{III}(HL2)] and [Cu^{III}(L2)]. Shaded areas under the VtC XES spectra represent the "double ionization" region (above 8979 and 8980 eV for d^9 and d^8 Cu, respectively), signifying energies far above the Fermi level of the complexes. The double ionization region produced emission features through multi-electron processes and therefore can have significant overlap with the absorption features from XAS.

The effect of Cu oxidation/reduction, ligand protonation and geometry

When the CuN_2S_2 first coordination spheres are held at a near ideal square planar geometry (*trans* angles fixed at 175°) the calculated VtC spectra look almost identical to that of the fully relaxed geometry optimized structures. Decreasing the *trans* CuN_2S_2 angles to 145°, to simulate a large tetrahedral distortion, results in only a small change in the intensity and energy of the VtC emission features, which would likely be unresolvable in our experimental set-up. This effectively shows that the VtC spectra of these complexes are not significantly sensitive to tetrahedral distortions of ~30°.

Table S2 Bond metrics from DFT optimized structures of [Cu^{II}(HL2)], [Cu^{III}(HL2)]$^+$, [Cu^{III}(L2)] and [Cu^{II}(L2)]$^-$.

	**[Cu^{II}(HL2)]**	**[Cu^{III}(HL2)]$^+$**	**[Cu^{III}(L2)]**	**[Cu^{II}(L2)]$^-$**
Lengths [Å]				
Cu-S (av)	2.27	2.19	2.20	2.29
Cu-N (av)	2.00	1.94	1.92	1.98
Angles [°]				

S1-Cu-S2	93.28	85.35	91.52	93.68
S1-Cu-N1	86.33	88.30	88.39	85.86
N1-Cu-N2	94.72	95.58	96.93	94.63
N2-Cu-S2	86.34	88.30	88.39	85.86
N1-Cu-S2	173.73	171.43	162.43	178.45
N2-Cu-S1	173.77	171.41	162.43	178.45
τ_4'	0.031	0.122	0.228	0.022

Table S3 Contribution of Cu d-/p-orbitals and ligand s-/p-orbitals to the calculated VtC spectra. All values are taken from the Löwdin MO population analysis using canonical orbitals.

Complex	Cu d [%]	Cu p [%]	Ligand s [%]	Ligand p [%]
$[Cu^{II}(HL1)]$ [a]	9.2 (11.9)	3.4 (3.0)	13.6 (13.4)	66.6 (64.5)
$[Cu^{II}(HL2)]$ [a]	9.9 (10.8)	2.8 (2.1)	12.4 (12.0)	67.0 (66.3)
$[Cu^{III}(HL2)]^{+}$	8.2	2.5	15.2	67.4
$[Cu^{III}(L2)]$	10.2	2.8	11.8	68.6
$[Cu^{III}(L2)]^{-}$ [a]	8.3 (6.7)	3.1 (3.3)	10.7 (10.3)	70.9 (72.8)

[a] For Cu(II) complexes values outside parentheses correspond to the *α*-spin manifold and values within parentheses correspond to the *β*-spin manifold.[b]

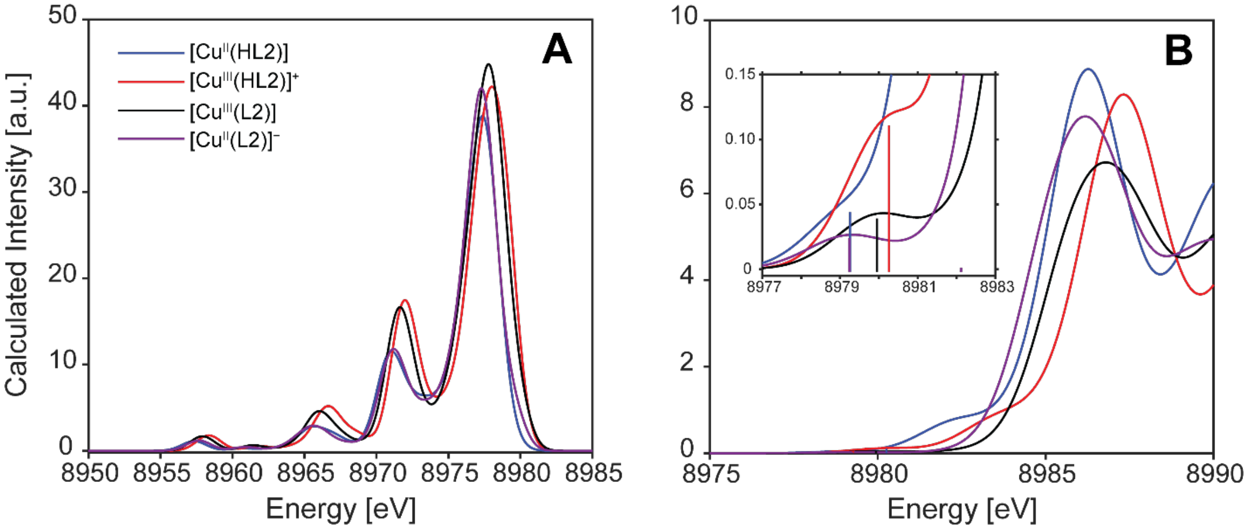

Figure S10 A) DFT calculated Cu Kβ VtC XES and **B**) TDDFT calculated Cu K-edge XAS for $[Cu^{II}(HL2)]$, $[Cu^{III}(L2)]$ and their *in silico* generated one-electron oxidation and reduction products.

Example Orca Input Files

Geometry Optimization

```
! UKS RIJK BP86 def2-TZVP AutoAux opt TightSCF Grid4 FinalGrid7 SlowConv NormalPrint

%pal
nprocs 12
end

%maxcore 3000

%scf
MaxIter 700
End

*xyzfile charge spin-multiplicity xyz_filename.xyz
```

X-ray Absorption Spectroscopy

```
! UKS RIJCOSX TPSSh ZORA ZORA-def2-TZVP D3BJ AutoAux TightSCF SlowConv Grid4 FinalGrid7
LargePrint UNO

%pal
nprocs 12
end

%scf
Maxiter 700
end

%maxcore 3000

%tddft
NRoots 200
MaxDim 900
OrbWin[0] = 0, 0, -1, -1
OrbWin[1] = 0, 0, -1, -1
Doquad true
end

*xyzfile charge spin_multiplicity xyz_filename.xyz
```

X-ray Emission Spectroscopy

```
! UKS RIJCOSX TPSSh ZORA ZORA-def2-TZVP D3BJ AutoAux Grid4 FinalGrid7 SlowConv TightSCF LargePrint

%pal
nprocs 12
end

%scf
MaxIter 700
end

%maxcore 3000

%xes
CoreOrb 0,0
OrbOp 0,1
CoreOrbSOC 0,1
DoSOC true
end

*xyzfile charge spin_multiplicity xyz_filename.xyz
```

Broken symmetry

```
! UKS RIJCOSX TPSSh ZORA ZORA-def2-TZVP D3BJ AutoAux Grid4 FinalGrid7 SlowConv TightSCF LargePrint

%maxcore 3000

%scf
BrokenSym 1,1
MaxIter 1200
end

*xyzfile charge spin_multiplicity xyz_filename.xyz
```

Vibrational Frequencies

```
! UKS RIJCOSX TPSSh def2-TZVP D3BJ AutoAux Grid4 FinalGrid7 SlowConv TightSCF LargePrint

%pal
nprocs 12
end

%maxcore 3000

*xyzfile charge spin_multiplicity xyz_filename.xyz
```

XYZ Coordinates for Orca Calculations

[Cu^{II}(HL1)] molecule A

Charge = 0, Spin Multiplicity = 2

Cu	-0.01565617833576	-0.02429052501548	-0.00039722820186
N	-1.40737718561208	2.57165120054794	-0.04910430412958
N	-0.15712050806751	1.98662979387653	-0.03825330071470
N	2.00134656567457	-0.05711184719572	0.00067444564410
N	2.65320875928829	-1.27354464349131	0.02415401600256
S	-2.28782387010189	-0.02011461222097	-0.00147986096349
S	0.11266279917015	-2.29373871388410	0.04165328226394
S	-4.04483564211121	2.31315413751602	-0.04444917997877
S	2.53847973771827	-3.92063165389110	0.07430136001541
H	2.75687172954559	2.91833107082102	0.80677037696068
H	0.01863007632238	4.62168440725439	0.79017491323962
H	4.60681763871065	0.24319473990046	-0.88311042960678
H	-3.27582152256387	4.42191878578902	-0.98058722208077
H	4.61820677854191	-3.02010824891256	0.95670692881549
C	-2.39829203396194	1.70672451086277	-0.03309315427434
C	0.84030664216290	2.82370133363270	-0.05424097813484
C	2.27758605754902	2.39569316712274	-0.04630330583879
H	2.75653327135639	2.88510247286459	-0.91906537904702
C	2.78317876916938	0.98411669704635	-0.01921080869886
C	1.84317122440578	-2.30972119985768	0.04339153499071
C	0.61127036466894	4.30963165764274	-0.08239097508980
H	1.56144164358875	4.85889766681806	-0.09297679536237
H	0.01829927401690	4.58838670067367	-0.96592976012622
C	4.27961484737008	0.83606615259195	-0.01627425733658
H	4.77659033368756	1.81447186475916	-0.03526486967687
H	4.60673829729317	0.27734861949250	0.87299347344119
C	-3.81586659082444	4.11597179732503	-0.07749436808269
H	-4.82934199674054	4.53810590458231	-0.08513546941585
H	-3.27566738139259	4.45473427536554	0.81371277204965
C	4.32641617130993	-3.59401013105022	0.06994347593289
H	4.80301462447622	-4.58289612187417	0.08945305586734
H	4.62019243868499	-3.05456997109221	-0.83755710046339

[Cu^{II}(HL1)] molecule B

Charge = 0, Spin Multiplicity = 2

Cu	-0.02408579724524	-0.01660593168151	0.00144178479286
N	-1.45220530992183	2.55918771836596	0.00201057595470
N	-0.19350584430241	1.99150203727121	0.00142605795811
N	1.98949306513407	-0.02271470994542	0.00063479696216
N	2.65080943084272	-1.22694295566311	0.00064445791435
S	-2.29841632720331	-0.04498650187461	0.00337090993232
S	0.13539737634349	-2.28791593636616	0.00215046369092
S	-4.08612815021851	2.26538259466573	0.00358967467974
S	2.78982698117605	-3.78430280086167	0.00069786518791
H	2.70626511919293	2.94752523010236	-0.86311969583422
H	-0.05347143591507	4.61202232120974	0.87933831266118
H	4.58897282964724	0.32067720956303	-0.87763455381117

H	-3.34716789950665	4.39807633149348	-0.90174116590642
H	0.88820064088772	-5.00985204089312	-0.90046200502684
C	-2.43101475350833	1.68141745555319	0.00288762391292
C	0.79271349174307	2.84205385894625	0.00081295277134
C	2.23553248878928	2.43386638458779	0.00012749326658
H	2.70714142530128	2.94780336003329	0.86273130681248
C	2.75958041875658	1.02935442698093	0.00009994566854
C	1.86226887662378	-2.28209759066744	0.00140398359960
C	0.54273593373583	4.32497569548257	0.00078294435973
H	1.48526828803019	4.88709220634798	0.00023500284378
H	-0.05438002679128	4.61183436149343	-0.87721681311356
C	4.25698868687214	0.89535291075281	-0.00054226256094
H	4.74470128934767	1.87858704946633	-0.00091181764719
H	4.58974536939189	0.32095189856916	0.87643742209512
C	-3.88134869653047	4.07130767662860	-0.00253234527980
H	-4.90018301217208	4.48029413813569	-0.00202264492458
H	-3.34398079596251	4.40378469761144	0.89267859160381
C	1.51262496673009	-5.07138884155436	-0.00160480713474
H	2.06762689769207	-6.01903250407454	-0.00194452596573
H	0.88657784003958	-5.01144035567906	0.89623051553705

[Cu^{II}(HL2)]

Charge = 0, Spin Multiplicity = 2

Cu	-0.130460163	0.149019429	0.013668716
N	-1.557122970	-2.426954203	-0.026370032
N	-0.298888680	-1.858592487	0.021056476
N	1.882011086	0.158786759	0.100568363
N	2.547572837	1.369253272	0.118112764
S	-2.396723154	0.174815596	-0.162117648
S	0.018933463	2.416626442	0.082136225
S	-4.194164007	-2.115912893	-0.170795500
S	2.445185010	4.026326798	0.124545804
C	-2.529927390	-1.549837487	-0.111500168
C	0.686354112	-2.708201742	0.068781363
C	2.126944193	-2.296277571	0.128462567
H	2.562581439	-2.802274506	1.014238868
H	2.636857268	-2.814329722	-0.709415576
C	2.650567665	-0.891217890	0.141637909
C	1.749155531	2.411052773	0.113073332
C	0.440361281	-4.190897663	0.067823202
H	-0.150605859	-4.478771943	0.950502932
H	1.384342087	-4.750815023	0.068115425
H	-0.161021234	-4.478880053	-0.806456783
C	4.146626113	-0.762402841	0.206013527
H	4.522173516	-0.241384748	-0.687714785
H	4.627917458	-1.746129649	0.278379649
H	4.441075307	-0.141597148	1.064421365
C	-4.044046426	-3.941573548	-0.196693739
H	-3.360391307	-4.236526881	0.609453026
H	-5.059273620	-4.262933750	0.089715713
C	-3.638197493	-4.581254761	-1.506537227
C	-4.069404042	-4.087681731	-2.745336268
H	-4.663317245	-3.172239067	-2.779288003

C	-3.728555298	-4.742436945	-3.930882776
H	-4.070668566	-4.340897751	-4.886396077
C	-2.951740974	-5.904015307	-3.897881018
H	-2.685073473	-6.413956165	-4.825022544
C	-2.517102638	-6.405313327	-2.668258033
H	-1.908890771	-7.311037894	-2.629524398
C	-2.857003875	-5.746272070	-1.483944427
H	-2.515651594	-6.143609927	-0.524900818
C	4.249337160	3.733614639	0.247559902
H	4.524316990	2.990673521	-0.511881284
H	4.665139331	4.705135740	-0.067524580
C	4.793964787	3.341973265	1.604007474
C	5.896701978	2.477406438	1.672402845
H	6.311811786	2.064855971	0.749540058
C	6.471981205	2.144090509	2.901367366
H	7.330221947	1.470192551	2.933441078
C	5.947615510	2.669393848	4.085154484
H	6.391894537	2.408220330	5.047013472
C	4.847504160	3.529707468	4.027516705
H	4.428446686	3.943159575	4.946598015
C	4.276613482	3.863605061	2.797416458
H	3.407861335	4.523790704	2.760618535

$[Cu^{III}(HL2)]^{+}$

Charge = +1, Spin Multiplicity = 0

Cu	0.161845275	-0.169978401	0.031203517
N	-1.336051535	-2.615139412	0.175174466
N	-0.061034153	-2.095568916	0.119813646
N	2.101109448	-0.102781277	0.031016243
N	2.723555397	1.123207011	-0.059418960
S	-2.009165795	0.006146595	-0.177745326
S	0.151161117	2.015993156	0.126634880
S	-3.970737740	-2.079702619	0.130520680
S	2.400922125	3.790258223	-0.168151036
C	-2.272039182	-1.717300978	0.068364310
C	0.920060421	-2.953080675	0.167918386
C	2.361594413	-2.551581678	0.146293662
H	2.834447037	-3.018607474	1.033463697
H	2.832869399	-3.106617594	-0.689447164
C	2.878285297	-1.148871313	0.074566448
C	1.899951179	2.130652038	-0.043339095
C	0.648330297	-4.423797461	0.250955624
H	0.078909297	-4.650883033	1.164763677
H	1.579607917	-5.001579254	0.250069864
H	0.015860326	-4.737439612	-0.592409773
C	4.368329690	-0.997226775	0.053663340
H	4.683460898	-0.500770534	-0.876502070
H	4.868729915	-1.968957165	0.134095854
H	4.691406289	-0.343328391	0.876834485
C	-4.049965294	-3.908843043	-0.112860625
H	-3.420465269	-4.370415688	0.658197493
H	-5.104140345	-4.100587247	0.144729173
C	-3.714554560	-4.414060517	-1.489883082

C	-4.338625047	-3.896463151	-2.635572416
H	-5.047902072	-3.071215481	-2.542540915
C	-4.056737959	-4.426671662	-3.894231863
H	-4.550238504	-4.017092541	-4.776604955
C	-3.152468106	-5.486536856	-4.026006331
H	-2.939614148	-5.904343616	-5.010804556
C	-2.531794471	-6.012896267	-2.891286243
H	-1.836764929	-6.848760511	-2.985175745
C	-2.807887421	-5.475308810	-1.631137583
H	-2.337819354	-5.901092103	-0.741701088
C	4.217825550	3.737165524	0.157411380
H	4.660300018	3.037033796	-0.562242265
H	4.505171042	4.759365520	-0.137620242
C	4.634786221	3.429837465	1.570229475
C	5.619110950	2.456912584	1.800081468
H	6.048150040	1.917178689	0.952704965
C	6.078672087	2.201101776	3.094915807
H	6.856114831	1.453034764	3.257789175
C	5.549786884	2.909250932	4.175994516
H	5.906714166	2.712283575	5.187686431
C	4.566275586	3.880072431	3.955765134
H	4.155550151	4.441627405	4.795903580
C	4.114429880	4.141190678	2.662483964
H	3.348521221	4.902661887	2.500337925

[Cu^{III}(L2)]

Charge = 0, Spin Multiplicity = 0

Cu	0.000000000	0.000000000	0.000000000
N	0.144611569	-1.905369073	0.285329803
N	1.382298495	-2.457184085	0.436662792
S	2.208879460	0.084030786	-0.014239033
S	3.999890416	-2.091934706	0.851519360
C	-2.656443824	-1.057412661	-0.507809123
C	-4.132476277	-0.882705400	-0.750454482
H	-4.590641051	-0.296267267	0.059447608
H	-4.629085891	-1.858084411	-0.812054659
H	-4.308712040	-0.321107376	-1.678291471
C	-1.747498104	2.271064424	-0.353419766
C	-3.936710983	3.587892833	-1.509767769
H	-3.709686808	3.014500783	-2.417488772
H	-4.560143292	2.972108846	-0.849931706
C	-4.579631039	4.910664045	-1.823793799
C	-5.455778625	5.514120021	-0.908626632
H	-5.673097244	5.012015247	0.036843483
C	-6.050374346	6.743982212	-1.197342006
H	-6.733369986	7.198568989	-0.477812232
C	-5.773018075	7.389944471	-2.405441030
H	-6.238465975	8.350368677	-2.632567204
C	-4.898050149	6.800003077	-3.322184011
H	-4.678001502	7.298602639	-4.267604935
C	-4.304125395	5.570397383	-3.031357553
H	-3.618853841	5.112764417	-3.748452691
N	-1.861864661	0.031100634	-0.514360078

N	-2.505850155	1.231050178	-0.587379912
S	-0.127462235	2.187215372	0.292281188
S	-2.341546245	3.900825642	-0.632860446
C	-2.152888923	-2.349381619	-0.286639694
H	-2.886090480	-3.149313118	-0.384150056
C	-0.855395662	-2.775008224	0.040079089
C	-0.572613539	-4.251858187	0.124000723
H	0.146772159	-4.552535658	-0.651804580
H	-1.497271893	-4.827129463	-0.002217497
H	-0.111864437	-4.503865997	1.089163195
C	2.366712367	-1.595583774	0.435353571
C	3.735839182	-3.808492047	1.481018737
H	3.039759110	-3.748589557	2.327222435
H	3.264289277	-4.384776058	0.675546846
C	5.066099761	-4.382470363	1.883972800
C	5.856286241	-5.075352532	0.954014503
H	5.495088422	-5.200091991	-0.069336191
C	7.093086855	-5.605697006	1.325971781
H	7.694210030	-6.146236230	0.592957322
C	7.559012634	-5.446401596	2.634142894
H	8.524802784	-5.861971326	2.926014824
C	6.782135479	-4.753833827	3.567338026
H	7.139584650	-4.626736982	4.590523957
C	5.545843063	-4.223778026	3.193192804
H	4.941842233	-3.680868252	3.923727794

[Cu^{II}(L2)]$^{-}$

Charge = -1, Spin Multiplicity = 2

Cu	0.13556061588355	0.12161534731544	-0.00072671027129
N	0.08457397543900	-1.84363981672770	-0.22080425131402
N	1.26236483322330	-2.53920680826866	-0.40233310522132
S	2.41110050590545	-0.08318369724814	-0.18634801244044
S	3.93882031749398	-2.55803001400339	-0.60482702038415
C	-2.69991279472606	-0.70375354826131	0.17392440585021
C	-4.17397214930069	-0.40794220280019	0.33163122041711
H	-4.37548767523053	0.09723860207087	1.28768223954275
H	-4.75637862705412	-1.33742906138224	0.28472845665739
H	-4.52231028373549	0.27597930005736	-0.45694582759454
C	-1.50799490681876	2.53870611741688	0.39466534156362
C	-3.89113257408977	4.03035237387710	0.77634007385434
H	-4.35309392103111	4.75167826634324	0.08811136097107
H	-4.08009769673323	3.00743693221101	0.41469732334911
C	-4.40436739112438	4.22369059505513	2.17657681235399
C	-4.03121688677166	3.34281801592886	3.20733061128257
H	-3.34376610512654	2.52603214922584	2.97782668963003
C	-4.52334975516804	3.51194069506998	4.50098654145004
H	-4.22369116802287	2.81617728279343	5.28755145183917
C	-5.39369553208206	4.56875493006495	4.79646382721860
H	-5.77376656398473	4.70190642799815	5.81133264386221
C	-5.76713169025372	5.45351263864664	3.78280000350060
H	-6.44162471673034	6.28470040326481	4.00082532952989
C	-5.27560592272232	5.27917922115740	2.48486928252989
H	-5.57185660864527	5.97369026819401	1.69410851032455

N	-1.82227491806029	0.30944442783137	0.21138698549551
N	-2.37010329920379	1.56248104345830	0.39583209528306
S	0.20857257599937	2.40433814550889	0.19420510279609
S	-2.06315890559447	4.22081290110106	0.60817723024887
C	-2.31154962778810	-2.04340187467279	-0.01195525361039
H	-3.12793965083131	-2.76551623078211	-0.01623196971423
C	-1.02819801927315	-2.59141992464803	-0.19323725934226
C	-0.91318798280541	-4.08966403337612	-0.35823912349455
H	-0.42784856236305	-4.34602479847729	-1.31135747436360
H	-1.90718852716380	-4.55445953107485	-0.32246470806773
H	-0.28398147367656	-4.52328045886394	0.43370527637109
C	2.33625082924106	-1.80257672863048	-0.39396299238126
C	3.52737256079991	-4.34818047738125	-0.78007296238662
H	4.18480653794987	-4.89684726551792	-0.09155576394755
H	2.48789813305272	-4.41215634668855	-0.42148812594245
C	3.66017770875467	-4.87797497595660	-2.18126001558498
C	2.84049900000759	-4.39119326402313	-3.21501685575965
H	2.11899577050283	-3.60379287432772	-2.98742891104739
C	2.95012952008886	-4.89864433764718	-4.50914070530606
H	2.30285532432520	-4.50948478619451	-5.29793049793584
C	3.88578960833735	-5.89869024809760	-4.80210467353166
H	3.97272548370187	-6.29095343939571	-5.81733004641417
C	4.70976513970009	-6.38599946523532	-3.78552385926306
H	5.44709894447195	-7.16246345347892	-4.00154045588642
C	4.59485561820355	-5.87847360488383	-2.48713157283308
H	5.24143602002951	-6.26401347954519	-1.69418119588305

References

1 J. K. Bilyj, N. V. Silajew, G. R. Hanson, J. R. Harmer and P. V. Bernhardt, *Dalt. Trans.*, 2019, **48**, 15501–15514.

2 G. M. Sheldrick, *Acta Crystallogr. Sect. A Found. Crystallogr.*, 2015, **71**, 3–8.

3 C. F. Macrae, I. Sovago, S. J. Cottrell, P. T. A. Galek, P. McCabe, E. Pidcock, M. Platings, G. P. Shields, J. S. Stevens, M. Towler and P. A. Wood, *J. Appl. Crystallogr.*, 2020, **53**, 226–235.

4 B. Ravel and M. Newville, *J. Synchrotron Radiat.*, 2005, **12**, 537–541.

5 N. Levin, S. Peredkov, T. Weyhermüller, O. Rüdiger, N. B. Pereira, D. Grötzsch, A. Kalinko and S. DeBeer, *Inorg. Chem.*, 2020, **59**, 8272–8283.

6 B. L. Geoghegan, Y. Liu, S. Peredkov, S. Dechert, F. Meyer, S. DeBeer and G. E. Cutsail, *J. Am. Chem. Soc.*, 2022, **144**, 2520–2534.

7 F. Neese, *Wiley Interdiscip. Rev. Comput. Mol. Sci.*, 2012, **2**, 73–78.

8 F. Neese, *Wiley Interdiscip. Rev. Comput. Mol. Sci.*, 2018, **8**, 4–9.

9 C. Lee, W. Yang and R. G. Parr, *Phys. Rev. B*, 1988, **37**, 785–788.

10 F. Weigend and R. Ahlrichs, *Phys. Chem. Chem. Phys.*, 2005, **7**, 3297–3305.

11 J. P. Perdew, J. Tao, V. N. Staroverov and G. E. Scuseria, *J. Chem. Phys.*, 2004, **120**, 6898–6911.

12 K. P. Jensen, *Inorg. Chem.*, 2008, **47**, 10357–10365.

13 G. L. Stoychev, A. A. Auer and F. Neese, *J. Chem. Theory Comput.*, 2017, **13**, 554–562.

14 F. Neese, F. Wennmohs, A. Hansen and U. Becker, *Chem. Phys.*, 2009, **356**, 98–109.

15 E. Van Lenthe, P. E. S. Wormer and A. Van Der Avoird, *J. Chem. Phys.*, 1997, **107**, 2488–2498.

16 D. A. Pantazis, X. Y. Chen, C. R. Landis and F. Neese, *J. Chem. Theory Comput.*, 2008, **4**, 908–919.

17 A. W. Addison, T. N. Rao, J. Reedijk, J. van Rijn and G. C. Verschoor, *J. Chem. Soc., Dalt. Trans.*, 1984, 1349–1356.

18 A. Okuniewski, D. Rosiak, J. Chojnacki and B. Becker, *Polyhedron*, 2015, **90**, 47–57.
